# Perceived Biological Bases of Sexual Orientation and Sexual Prejudice: The Moderating Role of Gender and Religious Beliefs

**DOI:** 10.1007/s10508-024-03070-6

**Published:** 2024-12-23

**Authors:** Juan M. Falomir-Pichastor, Dan Confino, Joel R. Anderson, Yasin Koc

**Affiliations:** 1 Faculty of Psychology and Educational Sciences, University of Geneva, Boulevard du Pont d’Arve 40, 1205 Geneva, Switzerland; 2 Institute for Positive Psychology and Education, Australian Catholic University, Fitzroy, Australia; 3 Australian Research Centre in Sex, Health and Society, La Trobe University, Bundoora, Australia; 4 Faculty of Behavioural and Social Sciences, University of Groningen, Groningen, The Netherlands

**Keywords:** Sexual prejudice, Sexual orientation, Religiosity, Biological determinism, Intergroup differences

## Abstract

Prior correlational studies have shown that belief in the biological theory of sexual orientation (BTSO) is associated with more positive attitudes toward homosexuality. However, individuals often interpret scientific evidence in ways that align with their pre-existing beliefs and motivations. This research experimentally investigated whether gender and religiosity moderate heterosexual individuals’ responses to scientific evidence either supporting or refuting BTSO. In two studies, heterosexual men (Study 1, *N* = 118) and both men and women (Study 2, *N* = 280) reported their religiosity and were exposed to evidence suggesting either biological differences or similarities between heterosexual and gay individuals. Results showed that, in the biological differences condition, heterosexual women and less religious men were more likely to perceive homosexuality as a natural biological variation and expressed more positive attitudes toward it. In contrast, more religious men interpreted the same evidence as indicating a biological anomaly in gay individuals and displayed more negative attitudes toward homosexuality.

**Supplementary Information:**

The online version contains supplementary material available at 10.1007/s10508-024-03070-6.

## Introduction

Issues surrounding sexual orientation remain socially and scientifically contentious, often centered on the debate between biological (nature) and social (nurture) determinants. While no single causal theory has gained widespread scientific consensus, evidence generally supports biological influences (e.g., genetics and hormones) over social or environmental factors, such as early experiences or cultural acceptance (Bailey et al., 2016). This evidence has shaped social, political and moral debates (Bailey et al., 2016; Cook, 2021; Halley, 1994; Shostak et al., 2009), raising a critical question: does awareness of biological evidence influence attitudes toward nonheterosexual orientations and individuals?

Correlational studies consistently show a link between lay beliefs in biological differences between heterosexual and gay individuals—referred to as the biological theory of sexual orientation (BTSO)—and more positive attitudes toward homosexuality. However, experimental research on this topic is limited and inconclusive, suggesting that individuals tend to interpret BTSO evidence in ways that align with their pre-existing beliefs and motivations. To deepen our understanding of the relationship between BTSO and sexual prejudice, this research adopts an experimental approach to investigate whether religiosity and gender moderate how individuals interpret and respond to scientific evidence supporting or refuting BTSO.

### Perceived Controllability and Sexual Prejudice

Attribution theory posits that perceptions of controllability influence attitudes: behaviors deemed controllable elicit more negative judgments, while those attributed to biological or external factors are associated with more positive attitudes (Weiner, 1993). Consistent with this, correlational studies have linked biological attributions of sexual orientation to reduced sexual prejudice, as such attributions imply that sexual orientation is beyond individual control (Ernulf et al., 1989; Frias-Navarro et al., 2015; Haider-Markel & Joslyn, 2008; Hewitt & Moore, 2002; Jayaratne et al., 2006; Sloane & Robillard, 2018; Tygart, 2000; Whitley, 1990).

This explanation, however, faces challenges, as most supporting evidence is correlational, raising concerns about causality (Hegarty, 2010; Hegarty & Golden, 2008). Notably, less prejudiced individuals are more likely to endorse biological explanations (Hegarty, 2002). Experimental studies on responses to BTSO evidence are limited, and yield inconsistent findings (Falomir-Pichastor & Mugny, 2009; Hegarty & Golden, 2008; Oldham & Kasser, 1999; Piskur & Degelman, 1992; Pratarelli & Donaldson, 1997). Beyond sexual orientation, biological explanations often correlate with negative attitudes in domains such as mental disorders or other stigmatized groups (Keller, 2005; Khan et al., 2018; Kvaale et al., 2013; Park et al., 2018; Phelan, 2005). Thus, attribution theory alone may not fully explain responses to BTSO evidence. Instead, biological explanations may serve distinct functions based on context and individual motivations (Drescher, 2015; Falomir-Pichastor & Mugny, 2009; Halley, 1994; Haslam & Levy, 2006; Hegarty, 2002; Kvaale et al., 2013; Morton & Postmes, 2009; Shostak et al., 2009; Verkuyten, 2003).

### Biased Assimilation of Biological Explanations for Sexual Orientation

Biological explanations for intergroup differences are a component of psychological essentialism, which posits that group members share an inherent “essence” that determines their attributes and behaviors (Haslam & Levy, 2006; Yzerbyt et al., 2001). Beliefs about the biological basis of sexual orientation reflect two dimensions of essentialism: naturalness/immutability, associated with positive attitudes toward homosexuality, and discreteness/entitativity, linked to stereotyping and prejudice (Haslam & Levy, 2006; Haslam et al., 2002; Hegarty, 2002). Consequently, evidence supporting the BTSO may lead to viewing homosexuality as either a natural biological variation or a deviant biological anomaly. For instance, evidence supporting the biological determinism of transgender identity increases perceptions of both its naturalness and discreteness (Ching & Chen, 2022). Similarly, endorsement of the BTSO correlates with positive attitudes when genes are seen as determining sexual orientation but with negative attitudes when genes are linked to specific “gay traits” (Khan et al., 2018).

Why do individuals interpret the same scientific evidence differently? Motivated reasoning suggests that interpretations of evidence often align with individuals’ pre-existing beliefs and motivations (Kunda, 1990; Lord et al., 1979). Research supports this functional role of biological explanations in shaping sexual prejudice. Highly identified LGB individuals strategically adopt essentialist beliefs to justify their attitudes and respond to perceived threats (Morton & Postmes, 2009). Exposure to BTSO evidence reduces prejudice among less prejudiced individuals but reinforces it among those with stronger prejudicial attitudes (Boysen & Vogel, 2007). For individuals high in right-wing authoritarianism (RWA), evidence of biological determinism increases negative attitudes and discreteness beliefs about transgender identity, while having no effect on those low in RWA (Ching & Chen, 2022; Ching et al., 2020).

Despite these findings, further research is needed to clarify the factors moderating how individuals interpret BTSO evidence. The present research focuses on two key predictors of sexual prejudice that may influence such interpretations: gender and religiosity.

#### The Moderating Role of Gender

Research consistently shows that heterosexual men display greater sexual prejudice, particularly toward gay men, compared to heterosexual women. This gap persists despite overall improvements in attitudes toward sexual minorities (Kite et al., 2021; Whitley & Ægisdóttir, 2000). Gender-role socialization encourages heterosexual men to define their identities in opposition to gay men, motivating them to create psychological distance and react defensively when their masculinity or heterosexuality is perceived as threatened (Bosson et al., 2005; Carnaghi et al., 2011; Herek, 1986; Herek & McLemore, 2013; Talley & Bettencourt, 2008; Vandello et al., 2008). In contrast, while heterosexual women may occasionally feel threatened by lesbians (Kite et al., 2021), they are generally less affected by such identity threats (Bosson & Michniewicz, 2013; Bosson et al., 2005). Consequently, men’s heightened sexual prejudice stems from a stronger motivation to differentiate themselves from gay men on both personal and group levels.

In line with this reasoning, evidence supporting the BTSO may satisfy heterosexual men’s need for distinctiveness, thereby reducing reactive prejudice. For instance, heterosexual men are more likely to endorse the BTSO when their group distinctiveness is threatened by egalitarian norms (Falomir‐Pichastor & Hegarty, 2014). Exposure to BTSO evidence has also been shown to reduce sexual prejudice among heterosexual men, particularly those motivated to distance themselves from gay men (Falomir-Pichastor & Mugny, 2009; Falomir-Pichastor et al., 2017; Iacoviello et al., 2020). Importantly, this effect is not observed among heterosexual women (Falomir‐Pichastor & Hegarty, 2014; Falomir-Pichastor et al., 2017) and is unrelated to perceived controllability of sexual orientation. These findings suggest that BTSO evidence fulfills men’s ego-defensive or boundary-reinforcement needs (Haslam & Levy, 2006).

In sum, heterosexual men interpret BTSO evidence based on their pre-existing differentiation motivations, viewing it either positively (as a natural biological expression) or negatively (as deviant biology). To better understand these opposing reactions, this research examines religiosity as a potential moderator.

#### The Moderating Role of Religiosity

Religiosity is a multidimensional construct shaped by diverse religious traditions, theological perspectives, and cultural contexts (Holdcroft, 2006; Saroglou, 2019). In this research, we define religiosity as the intensity, salience, and centrality of an individual’s religious beliefs and feelings (Huber & Huber, 2012), independent of specific religious affiliations.

Religiosity consistently correlates with sexual prejudice across major world religions (Etengoff & Lefevor, 2021). Many religions have historically framed heterosexuality as natural while labeling same-sex behaviors as sinful or unnatural deviations (Herek et al., 2007; Layman & Carmines, 1997; Thomas & Olson, 2012; Tygart, 2000). In Western contexts, where Christianity predominates, same-sex behavior has been stigmatized as incompatible with Christian teachings (Herek et al., 2007), and religious individuals tend to exhibit higher sexual prejudice compared to non-religious individuals (Anderson & Koc, 2015; Finlay & Walther, 2003; Moore et al., 2019). This association holds across dimensions of religiosity, including affiliation, participation, and beliefs like fundamentalism and intrinsic or extrinsic orientations (Anderson & Koc, 2015; Anderson et al., 2022; Jonathan, 2008; Kite et al., 2021; Van Der Toorn et al., 2017; Whitley, 2009). Sexual prejudice may also serve psychological functions, helping individuals affirm their moral identity by rejecting those perceived as violating religious values (Herek, 1987; Herek et al., 2007).

Religiosity may also shape interpretations of evidence supporting the BTSO. Strongly religious individuals may process such evidence through a self-serving lens (Kunda, 1990; Lord et al., 1979). For instance, evangelical elites construct moral narratives to counteract BTSO attribution effects (Haider-Markel & Joslyn, 2008; Thomas & Whitehead, 2015; Whitehead, 2010). However, no experimental studies have directly investigated whether religiosity moderates interpretations to BTSO evidence. This research addresses this gap by examining whether religiosity influences how heterosexual men interpret BTSO evidence.

We propose that heterosexual men, driven by a strong motivation to differentiate themselves from gay men, are influenced by religiosity when interpreting BTSO evidence. Men with strong religiosity may view BTSO evidence as emphasizing fundamental intergroup differences, thereby interpreting homosexuality as a biological anomaly. In contrast, men with lower religiosity may interpret such evidence as legitimizing natural variation in human sexuality. While exposure to BTSO evidence may reduce prejudice overall, it is less effective among highly religious heterosexual men, who align their interpretations with pre-existing religious beliefs. Heterosexual women, being less motivated by ingroup distinctiveness, are less likely to interpret BTSO evidence as reflecting fundamental differences, even if religiosity predicts their sexual prejudice.

### Overview and Hypotheses

We conducted two studies to test the main hypothesis that religiosity moderates the effect of exposure to scientific evidence supporting the BTSO on heterosexual men’s sexual prejudice. Both studies were conducted in Switzerland, where Christianity (Catholic and Protestant) is the dominant religion. Study 1 focused on a sample of heterosexual men, while Study 2 expanded the sample to include both heterosexual men and women to examine whether the investigated processes are specific to men. Religiosity was measured differently in each study: in Study 1, through self-identification as a believer, and in Study 2, through the centrality of religiosity. In both studies, we manipulated scientific evidence by presenting evidence either supporting the BTSO (biological differences condition) or refuting it (biological similarities condition). Study 2 also included a control condition where no information about the biological determinism of sexual orientation was provided. The main dependent variable in both studies was participants’ attitude toward homosexuality.

We hypothesized that exposure to scientific evidence supporting the BTSO would lead to more positive attitudes toward homosexuality among heterosexual men with lower religiosity, compared to exposure to evidence refuting the BTSO or no exposure. Conversely, among heterosexual men with higher religiosity, such exposure might not improve, and could even worsen, attitudes toward homosexuality. We expected this effect to be more pronounced, or exclusive, to heterosexual men, as heterosexual women generally show lower sexual prejudice and are less influenced by intergroup differentiation motives.

To deepen our understanding of the underlying processes, we included additional exploratory measures: perceived control over sexual orientation (Studies 1 and 2) and the perception of homosexuality as either a biological anomaly or a natural expression of sexuality (Study 2; see supplementary material for additional materials). Based on our rationale, we did not expect scientific evidence supporting the BTSO to influence perceived control over sexual orientation. For the perception of homosexuality as an anomaly versus a natural expression, we anticipated results consistent with the main hypothesis: exposure to BTSO evidence would likely reinforce the perception of homosexuality as a natural expression among heterosexual men with lower religiosity, but as a biological anomaly among heterosexual men with higher religiosity. No significant effects were expected for female participants.

## Study 1

### Method

#### Participants and Procedure^1^

This study was conducted as part of a master’s research, and the sample size was determined based on requirements of the social psychology unit for master’s research projects, which was at least 150 participants. Participants were male volunteers recruited through social networks to participate in a questionnaire-based study. Out of the initial 150 participants, 37 were excluded because they did not identify as men (*n* = 5) or as heterosexual (*n* = 35; see Measures: Sexual orientation). The final sample consisted of 118 heterosexual men with a mean age of 27.71 years (*SD* = 9.43; 68 students). Among them, 67 identified as atheist or had no religious affiliation, 6 as agnostic, 28 as Christians (2 Protestants, 19 Catholics, and the other 7 did not provide their denomination), 5 as Muslims, and 12 did not provide any information regarding their affiliation. Given that this study involved a continuous factor (religiosity) and a dichotomous variable (experimental induction of the biological bases of sexual orientation), we used a 2 × 2 experimental design as a proxy to conduct a sensitivity power analysis using G*Power for an ANOVA with four groups (α = 0.05, two-tailed, and a power of 0.80). This analysis confirmed that our final sample size was sufficiently powered to detect a medium effect size (*f* = 0.26). Participants were randomly assigned to one of the two experimental conditions (biological bases: differences, similarity).

#### Measures

**Religiosity**: To assess the religious beliefs, we used one single item at the end of the questionnaire. Participants were asked to describe their faith on a continuum ranging from 1 “*weak religious beliefs*” to 7 “*strong religious beliefs*” (*M* = 2.70, *SD* = 2.00).

**Biological bases of sexual orientation**: The evidence in support of the biological bases of sexual orientation was manipulated as in previous research (Falomir-Pichastor & Mugny, 2009). The material was sex-specific, so that the participants received information about the biological bases of male sexual orientation. Specifically, participants read a text that summarized scientific evidence ostensibly based on different studies, in which heterosexual men were compared with gay men with respect to genes, level of mother’s androgen during pregnancy, and the weight of the part of the hypothalamus that is responsible for sexual orientation. In the biological differences condition, the results of these studies highlighted the existence of biological differences between heterosexual and gay men, and then highlighted that sexual orientation is determined biologically—i.e., that gay and heterosexual men are biologically different, and thus not controllable. In the biological similarities condition, the results emphasized that heterosexual and gay men are biologically similar, indicating the lack of scientific evidence for a biological determinism of sexual orientation, and then highlighted that sexual orientation is controllable or a choice.

#### Dependent Variables

**Manipulation check**: We tested whether the manipulation of the biological bases of sexual orientation worked as expected through a single item: “Homosexuality is biologically determined” (1 “*not at all*” and 7 “*absolutely*”; *M* = 3.19, *SD* = 1.98) (Falomir-Pichastor & Mugny, 2009).

**Attitude toward homosexuality:** In this study, we used two scales assessing participants attitude toward gay people. First, we used the 16-item scale to measure participants’ positive Attitude Toward Homosexuality (ATH; e.g., “Gay couples should have the right to marry”; 1 = “*strongly disagree*” and 7 = “*strongly agree*”) (Anderson et al., 2018). An overall score measuring positive attitudes was computed by averaging the answers to all items after reverse-coding appropriate items (*M* = 4.48, *SD* = 1.29, α = 0.89). Second, we also included the 20-item scale assessing negative Attitudes Toward Lesbians and Gay Men (ATLG) (Herek, 1988), which is originally composed of two subscales: attitudes toward lesbians (ATL; *M* = 2.55, *SD* = 1.23, *α* = 0.87; e.g., “Female homosexuality is a sin”) and toward gay men (ATG; *M* = 3.03, *SD* = 1.46, *α* = 0.82; e.g., “Male homosexuality is a perversion”). However, both subscales were strongly correlated,* r*(118) = 0.76, *p* < 0.001, and they cannot actually be compared to each other given that each subscale includes different items. Therefore, we also computed an overall score of ATLG (*M* = 2.79, *SD* = 1.27, α = 0.91).

**Perceived controllability**: We measured perceived control over sexual orientation through 3 items (Falomir-Pichastor et al., 2017; Iacoviello et al., 2020): “Gay people, at some point in their life, decide upon their sexual orientation voluntarily”, “Gay people have the possibility to change their sexual orientation”, and “Gay people are personally responsible for their sexual orientation” (1 “*strongly disagree*” and 7 “*strongly agree*”). Scores for these items were averaged to form a reliable measure of perceived controllability (*M* = 3.32, *SD* = 1.85, α = 0.85).

**Sexual orientation:** At the end of the study, participants responded to several demographic items including three questions about their sexual orientation: they defined themselves as “heterosexual”, “bisexual”, or “homosexual”, indicated previous sexual relations with a person of the same-sex (“yes” vs. “no”), and indicated whether they felt attracted to people of the same-sex (on a scale ranging from 1 “*never*” to 7 “*frequently*”). If participants defined themselves as heterosexual, reported not having had sexual relationships with a same-sex person, and scored below the mid-point of the attraction item scale (4), then they were categorized as heterosexual and were retained for the analyses (Falomir‐Pichastor & Hegarty, 2014; Falomir-Pichastor & Mugny, 2009).

## Results

Correlations between all the measures are shown in Table 1. Unless otherwise indicated, we conducted a ANCOVA in which we introduced participants’ religiosity (standardized scores), the experimental condition (biological bases: -1 = differences, + 1 = similarities) and the interaction between these two factors as covariates.

**Table 1:** Correlations between variables (N = 118; Study 1)

	ATH	ATLG	PC
Religiosity	−0.161	0.124	0.043
Positive Attitude Toward Homosexuality (ATH)		−0.859^**^	−0.317^**^
Attitude toward Lesbians and Gay men (ATLG)			0.308^**^
Perceived Control (PC)			–

^*^ *p* < .05, ** *p* < .01 (2-tailed)

### Manipulation Check

The main analysis showed that the biological bases’ main effect was significant, *F*(1, 114) = 4.80, *p* = 0.030, η^2^_p_ = 0.040. Participants endorsed the belief that sexual orientation is biologically determined more strongly in the biological differences condition (*M* = 3.58, *SD* = 1.93) than in the biological similarities condition (*M* = 2.79, *SD* = 1.97). The religious beliefs main effect, *F*(1, 114) = 0.08, *p* = 0.784, η^2^_p_ = 0.001, and the interaction effect, *F*(1, 114) = 0.49, *p* = 0.485, η^2^_p_ = 0.004, were not significant.

### Positive Attitudes Toward Homosexuality

**Fig. 1:**
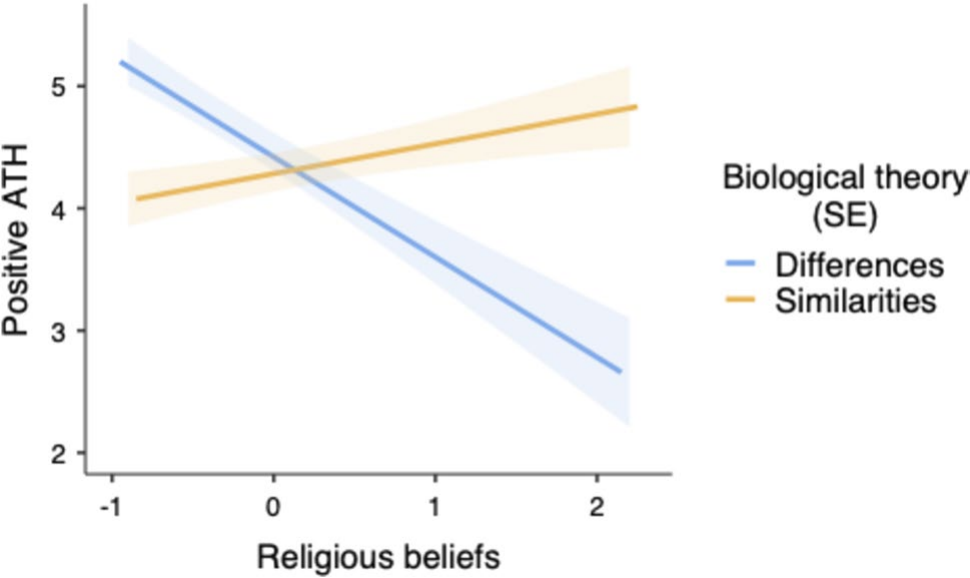
Predicted values for positive Attitudes Toward Homosexuality (ATH) as a function of religiosity and biological theory framing (Study 1)

The main analysis showed a significant main effect of religious beliefs, *F*(1, 114) = 6.43, *p* = 0.013, η^2^_p_ = 0.053, such that lower religiosity was related to more positive ATH (*B* = -0.28, *SE* = 0.11). More importantly, and as predicted, the biological bases × religiosity interaction was significant, *F*(1, 114) = 21.94, *p* < 0.001, η^2^_p_ = 0.161 (see Fig. 1). When religiosity was low (-1*SD*), attitude toward homosexuality was more positive in the differences condition than in the similarities condition, *t*(114) = 3.93, *p* < 0.001, η^2^_p_ = 0.120, whereas the reverse was observed when religiosity was high (+ 1*SD*), *t*(114) = 2.77, *p* = 0.006, η^2^_p_ = 0.063. We also decomposed the interaction in the reverse direction. Religiosity was related to less positive attitudes toward homosexuality in the biological differences condition (*B* = -0.82, *SE* = 0.17), *t*(114) = 4.68, *p* < 0.001, η^2^_p_ = 0.162, but not in the biological similarities condition (*B* = 0.24, *SE* = 0.14), *t*(114) = 1.68, *p* = 0.095, η^2^_p_ = 0.024.

### Negative Attitudes Toward Lesbians And Gay Men

We initially ran a mixed ANCOVA in which, in addition of the main between-subjects factors, the two subscales (ATG and ATL) were introduced as a within-subjects factor (a repeated measures analysis). This analysis showed a main effect of the subscales,* F*(1, 114) = 33.25, *p* < 0.001, η^2^_p_ = 0.226. As could be expected, attitudes were more negative for gay men than for lesbians. However, none of the interactions between the main independent variables and the within-subjects (subscale) factor was significant, *F*s < 3.34, *p*s > 0.07, which is consistent with the fact that both subscales were strongly correlated (see method section). Therefore, and for simplicity purposes, we only describe here the analysis for the overall negative ATLG score.

**Fig. 2:**
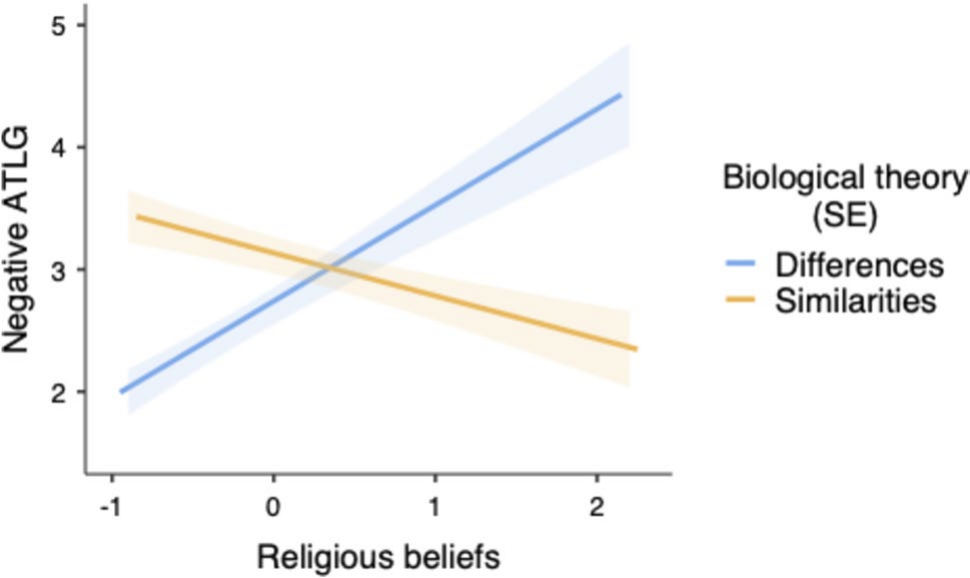
Predicted values for negative Attitudes Toward Lesbians and Gay men (ATLG) as a function of religiosity and biological theory framing (Study 1)

The main ANCOVA on negative ATLG scores revealed that the main effects of biological bases,* F*(1, 114) = 3.85, *p* = 0.052, η^2^_p_ = 0.033, and religiosity,* F*(1, 114) = 4.04, *p* = 0.047, η^2^_p_ = 0.034, were close to significance or significant, respectively. Participants tended to show more negative attitudes in the biological similarities condition (*M* = 3.04, *SD* = 1.18) than in the biological differences condition (*M* = 2.55, *SD* = 1.31), and religiosity was related to more negative attitudes (*B* = 0.21, *SE* = 0.10). More importantly, the biological bases × religiosity interaction was significant, *F*(1, 114) = 27.58, *p* < 0.001, η^2^_p_ = 0.195 (see Fig. 2). When religiosity was low (-1*SD*), attitudes were less negative in the differences condition than in the similarities condition, *t*(114) = 5.21, *p* < 0.001, η^2^_p_ = 0.193, whereas the reverse was observed when religiosity was high (+ 1*SD*), *t*(114) = 2.34, *p* = 0.021, η^2^_p_ = 0.046. Religiosity was strongly correlated with more negative attitudes in the differences condition (*B* = 0.78, *SE* = 0.16), *t*(114) = 4.71, *p* < 0.001, η^2^_p_ = 0.163, but with less negative attitudes in the similarities condition (*B* = -0.35, *SE* = 0.13), *t*(114) = 2.53, *p* = 0.012, η^2^_p_ = 0.053.^2^

### Perceived Controllability

**Fig. 3:**
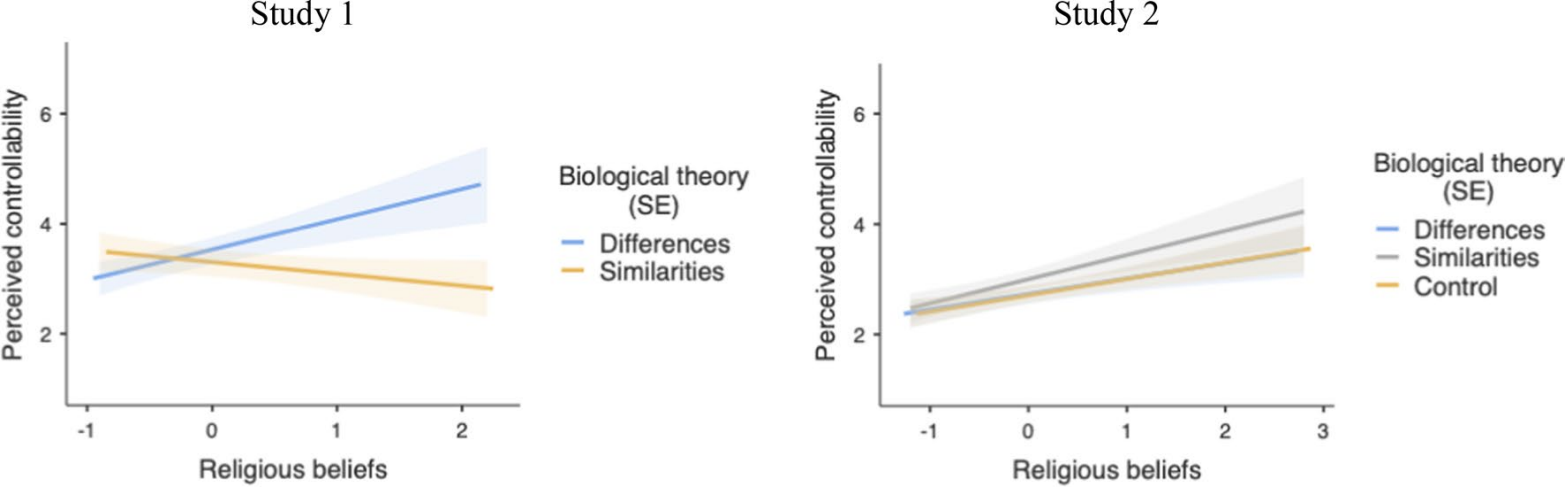
Predicted values for perceived controllability as a function of religiosity and biological theory framing (Study 1: left side; Study 2: right side)

The ANOVA only revealed a significant interaction effect, *F*(1, 114) = 4.73, *p* = 0.032, η_p_^2^ = 0.040 (Fig. 3, left side). When religiosity was low (-1*SD*), the experimental manipulation did not influence the perceived controllability, *t*(114) = 1.14, *p* = 0.254, η^2^_p_ = 0.011. However, when religiosity was high (+ 1*SD*), perceived controllability tended to be higher in the differences condition as compared to the similarities condition, although this effect did not reach significance, *t*(114) = 1.94, *p* = 0.054, η^2^_p_ = 0.032. Religious beliefs were related to greater perceived controllability in the biological differences condition (*B* = 0.55, *SE* = 0.48), *t*(114) = 2.03, *p* = 0.044, η^2^_p_ = 0.035, but not in the biological similarities condition (*B* = -0.21, *SE* = 0.22), *t*(114) = 0.95, *p* = 0.342, η^2^_p_ = 0.008.

## Discussion

The findings support the main hypothesis that religiosity moderates the effect of exposure to BTSO evidence on heterosexual men’s sexual prejudice. Among participants with low religiosity, sexual prejudice was lower in the biological differences condition compared to the biological similarities condition. Conversely, among participants with high religiosity, this pattern reversed: sexual prejudice was higher in the biological differences condition. This effect was consistent across two measures of sexual prejudice (ATH and ATLG).

Unexpectedly, participants with higher religiosity perceived sexual orientation as more controllable in the biological differences condition compared to the biological similarities condition. This finding contradicts attribution theory, which predicts that exposure to BTSO evidence should reduce perceived controllability. Instead, it suggests that perceived controllability may reflect motivated reasoning (Kunda, 1990), serving as a justification process (Hegarty, 2002).

## Study 2

We conducted a second study to replicate main findings and address limitations from Study 1. First, religiosity was assessed using a validated scale rather than a single-item measure. Second, the experimental manipulation was expanded to include a control condition where no information about the biological basis of sexual orientation was provided, alongside the biological differences and similarities conditions. Third, while Study 1 focused exclusively on heterosexual men—a group with stronger motivation to maintain psychological distance from gay individuals—Study 2 included both male and female participants to examine whether the observed processes were specific to men. Fourth, a new measure was introduced to assess whether the BTSO evidence was perceived as indicating homosexuality as a biological anomaly or as a natural expression of biology. Finally, given the consistent findings across two sexual prejudice measures in Study 1, Study 2 focused solely on the ATH scale (Anderson et al., 2018) to streamline the design and reduce questionnaire length.

### Method

#### Participants and Procedure

In this study, we aimed to recruit approximately 400 participants to ensure a minimum of 50 participants per experimental condition (*N* = 300) after exclusions, in a 2 (participant sex) × 3 (condition) experimental design (Simmons et al., 2013). Initial recruitment was conducted through social networks of a master’s student who acted as the experimenter for her thesis project. According to the requirements of the social psychology unit, master’s student was required to recruit 150 participants (see Study 1) and finally recruited 203 participants. To complete the sample, we used the French crowdsourcing platform FouleFactory, where participants received €3.5 for their participation. Within both recruitment methods, participants were asked to participate in a survey about different social issues by filling in an online questionnaire.

Of the initial 404 participants, we excluded 10 who did not provide consent, 12 who failed an attention check item, and 2 who were under 18 years old. Additionally, because the religiosity measure was adapted for a monotheistic concept of God, 10 polytheistic were excluded. Finally, 90 participants who did not identify as heterosexual were also excluded (see Study 1). The final sample included 280 participants (192 females and 88 males; 42 students; age range: 18–84 years, *M*_*age*_ = 41.28 years, *SD* = 14.63). The final sample included 100 Catholics, 14 Protestants, 9 Evangelists, 17 as Christians, 1 Jewish, 12 Muslims, and 127 Atheists. A sensitivity power analysis conducted using G*Power for a regression analysis with 11 predictors, assuming α = 0.05 (two-tailed) and a power of 0.80, indicated that the final sample was adequately powered to detect effects between small and medium sizes (*f*^*2*^ = 0.062). Participants were randomly assigned to one of the three conditions (*biological bases*: differences vs. similarities vs. control).

#### Measures

##### Religiosity

In this study, we assessed participants’ religiosity using the 15-item centrality of religiosity scale (Huber & Huber, 2012). For example, this scale includes items such as “How often do you think about religious issues?”, “To what extent do you believe that God or something divine exists?”, “How often do you take part in religious services?”, or “How often do you pray?” Participants rated the items on a 7-point scale (1 = *never/not agree at all*, 7 = *very often/totally agree*). We computed an average score, so that higher scores indicate stronger religiosity (*M* = 2.75, *SD* = 1.57; α = 0.96).

##### Biological bases of sexual orientation

Participants were randomly assigned to one of the three experimental conditions. The biological differences and similarities conditions were manipulated as in Study 1, and the information content was sex-matched: female participants received information on biological differences between heterosexual and lesbian women, while male participants received information on biological differences between heterosexual and gay men. Participants in the control condition did not receive any information about the biological basis of sexual orientation, but they did complete the manipulation check items (see dependent variables).

#### Dependent Variables

**Manipulation check**. To test the effectiveness of the experimental manipulation, in this study we included two items (Iacoviello et al., 2020): “Heterosexual and gay people are biologically different” and “Homosexuality is biologically determined (e.g., genetically, by hormones)”; α = 0.66, *M* = 3.14, *SD* = 1.74).

**Positive attitude toward homosexuality (ATH)**: Given the consistency of the results observed for the two attitude measures used in Study 1, in this study we only included the positive ATH scale (α = 0.93; *M* = 5.28, *SD* = 1.35), assessed as in Study 1.

**Perceived biological anomaly**. In the present study we created a bespoke 5-item scale assessing whether participants perceive same-sex/gender attraction is a result of a biological anomaly: “Homosexuality is biologically normal”, “There is something wrong with homosexuality”, “Homosexuality is the result of biological dysfunction”, “Gay people have a biological problem”, “Homosexuality is a natural expression of human sexuality” (α = 0.84; *M* = 2.52, *SD* = 1.48).

**Perceived controllability**. Perceived controllability of sexual orientation was assessed as in Study 1 (α = 0.74; *M* = 2.79, *SD* = 1.60).

### Results

Correlations between variables are presented in Table 2. We computed two contrasts from the three experimental conditions. Given that participants typically do not perceive sexual orientation as biologically determined (Costa et al., 2014), and that participants in control conditions perceive the biological bases of sexual orientation similarly to those in a biological similarities condition (Falomir-Pichastor & Mugny, 2009), we computed a first contrast (C1) by opposing the biologically-different condition (coded as + 2) to the other two conditions (each one coded as -1). The second residual contrast (C2) opposed the biological similarities condition (+ 1) to the control condition (-1), with the biological differences condition coded as 0. Dependent variables were analyzed using an ANCOVA, with these two contrasts, religiosity (standardized scores), participant’s sex (-1 = women and + 1 = men), and the interactions between these three factors (interactions including the two contrasts were not included) as covariates.

**Table 2:** Correlations between variables (N = 280; Study 2)

	ATH	PBA	PC
Religiosity	−0.296^**^	.316^**^	0.194^**^
Positive Attitude Toward Homosexuality (ATH)		−0.787^**^	−0.432^**^
Perceived Biological Anomaly (PBA)			0.441^**^
Perceived Control (PC)			–

^*^ *p* < .05, ** *p* < .01 (2-tailed)

#### Manipulation Check

As expected, the main analysis revealed a significant main effect of C1, *F*(1, 268) = 46.47, *p* < 0.001, η^2^_p_ = 0.148. Homosexuality was perceived as more biologically determined in the biological differences condition (*M* = 4.10, *SD* = 1.76) than in the biological similarities (*M* = 2.38, *SD* = 1.47) and control (*M* = 2.83, *SD* = 1.49) conditions. The effect of C2 was not significant, *F*(1, 268) = 2.40, *p* = 0.123, η^2^_p_ = 0.009, indicating that the perception of the biological bases of sexual orientation was similar in the biological similarities and control conditions. Finally, the analysis also revealed a main effect of participant’s sex, *F*(1, 268) = 7.56, *p* = 0.006, η^2^_p_ = 0.027. Male participants perceived to a greater extent that homosexuality is biologically determined (*M* = 3.38, *SD* = 1.79) compared to female participants (*M* = 3.02, *SD* = 1.71). No other main effect or interaction effects were significant.

#### Positive Attitudes Toward Homosexuality

The main effects of participant’s sex, *F*(1, 268) = 21.70, *p* < 0.001, η^2^_p_ = 0.075, and religiosity,* F*(1, 268) = 27.74, *p* < 0.001, η^2^_p_ = 0.094, were significant. In line with previous research, female participants (*M* = 5.49, *SD* = 1.26) displayed more positive attitudes toward homosexuality than male participants (*M* = 4.82, *SD* = 1.43). Furthermore, positive ATH decreased as religiosity increased (*B* = -0.47, *SE* = 0.09).

**Fig. 4:**
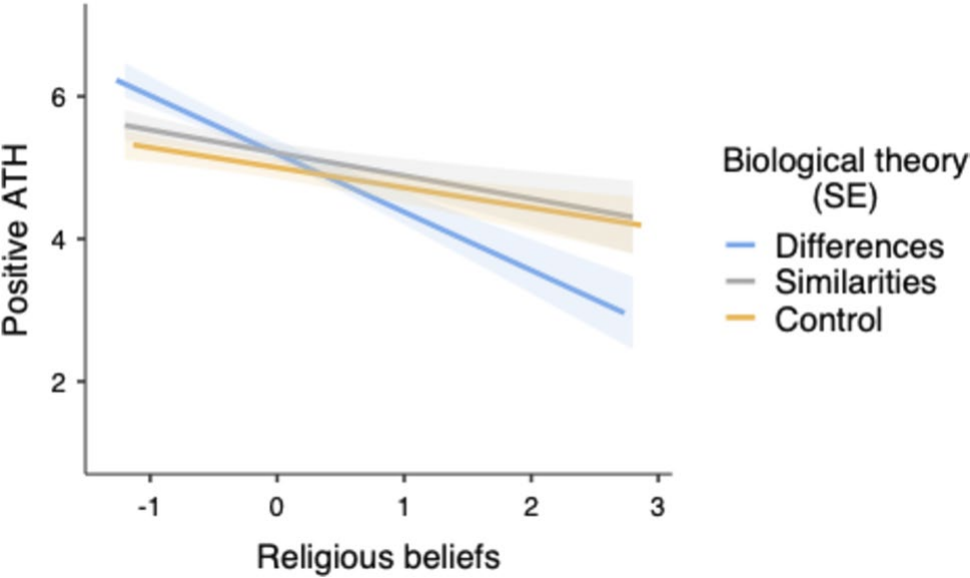
Predicted values for positive Attitudes Toward Homosexuality (ATH) as a function of religiosity and biological theory framing (Study 2)

The predicted interaction between religiosity and C1 was also significant,* F*(1, 268) = 6.62, *p* = 0.011, η^2^_p_ = 0.024 (see Fig. 4). When religiosity was low (-1*SD*), ATH were more positive in the differences condition than in the other two conditions (C1), *t*(268) = 2.64, *p* = 0.009, η^2^_p_ = 0.025. These two last conditions did not differ significantly (C2),* t*(268) = 0.99, *p* = 0.31, η^2^_p_ = 0.004. When religiosity was high (+ 1*SD*), the biological differences condition did not differ from the other two conditions (C1), *t*(268) = 1.30, *p* = 0.19, η^2^_p_ = 0.006, and those two conditions did not differ from each other (C2), *t*(268) = 0.59, *p* = 0.55, η^2^_p_ = 0.001. Furthermore, religiosity was more strongly associated with less positive ATH in the differences condition (*B* = -0.81, *SE* = 0.17), *t*(268) = 4.78, *p* < 0.001, η^2^_p_ = 0.079, compared to the similarities condition (*B* = -0.32, *SE* = 0.16), *t*(268) = 1.97, *p* = 0.049, η^2^_p_ = 0.014, and the control condition (*B* = -0.28, *SE* = 0.13), *t*(268) = 2.15, *p* = 0.032, η^2^_p_ = 0.017.

**Fig. 5:**
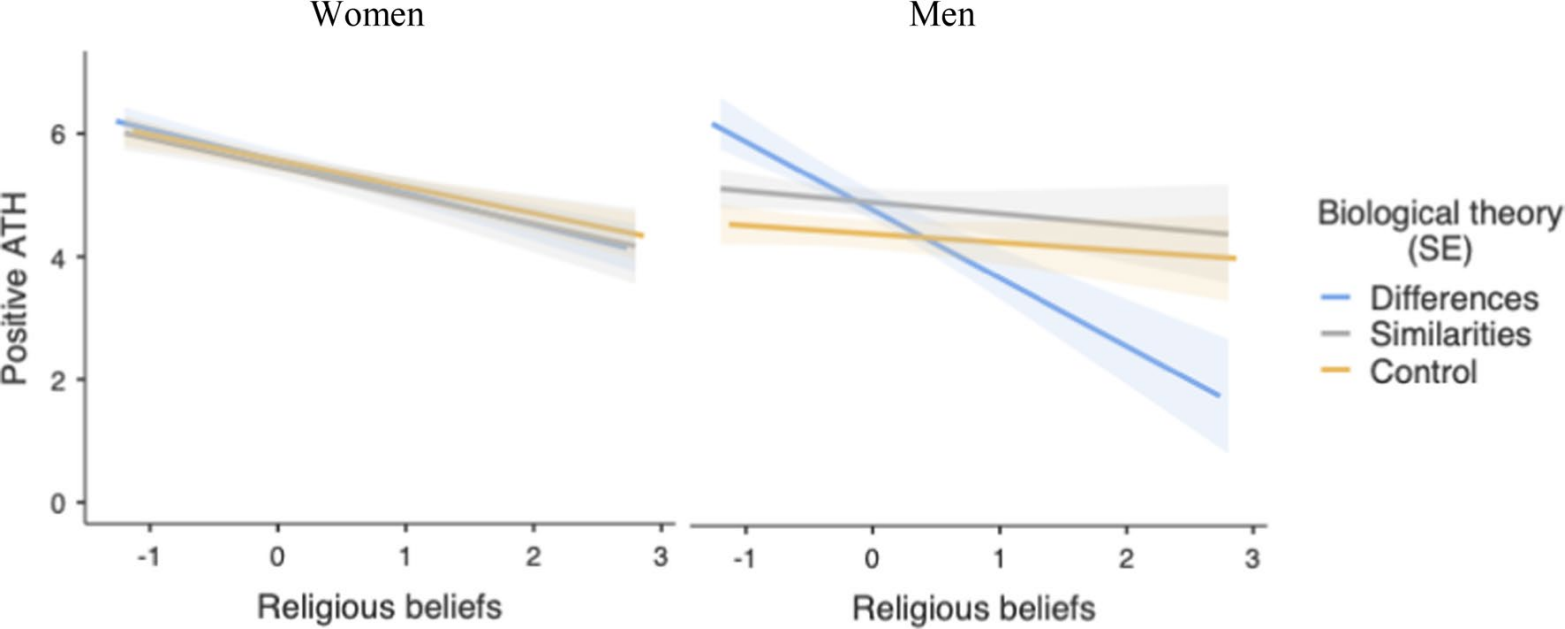
Predicted values for positive Attitudes Toward Homosexuality (ATH) as a function of religiosity, biological theory framing and participant’s gender (Study 2)

Finally, the ANCOVA also revealed a significant three-way interaction between C1, religiosity, and participant’s sex,* F*(1, 268) = 4.87, *p* = 0.028, η^2^_p_ = 0.018 (see Fig. 5). Among female participants, only the effect of religiosity was significant (*B* = -0.46, *SE* = 0.09), *t*(268) = 5.02, *p* < 0.001, η^2^_p_ = 0.086. Among male participants, the effect of religiosity was also significant (*B* = -0.48, *SE* = 0.15), *t*(268) = 3.12, *p* = 0.002, η^2^_p_ = 0.035, but this effect was moderated by C1, *t*(268) = 2.68, *p* = 0.008, η^2^_p_ = 0.024. When religiosity was low (-1*SD*), ATH were more positive in the differences condition than in the other two conditions (C1), *t*(268) = 2.73, *p* = 0.007, η^2^_p_ = 0.027. The similarities condition did not differ from the control condition (C2),* t*(268) = 1.38, *p* = 0.16, η^2^_p_ = 0.007. When religiosity was high (+ 1*SD*), ATH did not differ between the differences condition and the other two conditions (C1), *t*(268) = 1.50, *p* = 0.13, η^2^_p_ = 0.008, and those two conditions did not differ from each other (C2),* t*(268) = 0.92, *p* = 0.35, η^2^_p_ = 0.003. Furthermore, religiosity was related to less positive ATH in the differences condition (*B* = -1.11, *SE* = 0.31), *t*(268) = 3.56, *p* < 0.001, η^2^_p_ = 0.045, but not in the similarities condition (*B* = -0.18, *SE* = 0.25), *t*(268) = 0.72, *p* = 0.46, η^2^_p_ = 0.002, nor in the control condition (*B* = -0.13, *SE* = 0.22), *t*(268) = 0.62, *p* = 0.53, η^2^_p_ = 0.001.^3^

#### Perceived Biological Anomaly

The main ANCOVA revealed a significant main effect of participant’s sex, *F*(1, 268) = 24.04, *p* < 0.001, η^2^_p_ = 0.082. Male participants (*M* = 2.97, *SD* = 1.73) perceived homosexuality as a biological anomaly to a greater extent than female participants (*M* = 2.31, *SD* = 1.30). The main effect of religiosity was also significant, *F*(1, 268) = 37.43, *p* < 0.001, η^2^_p_ = 0.123, indicating that religiosity was related to a higher perception of homosexuality as a biological anomaly (*B* = 0.59, *SE* = 0.09).

**Fig. 6:**
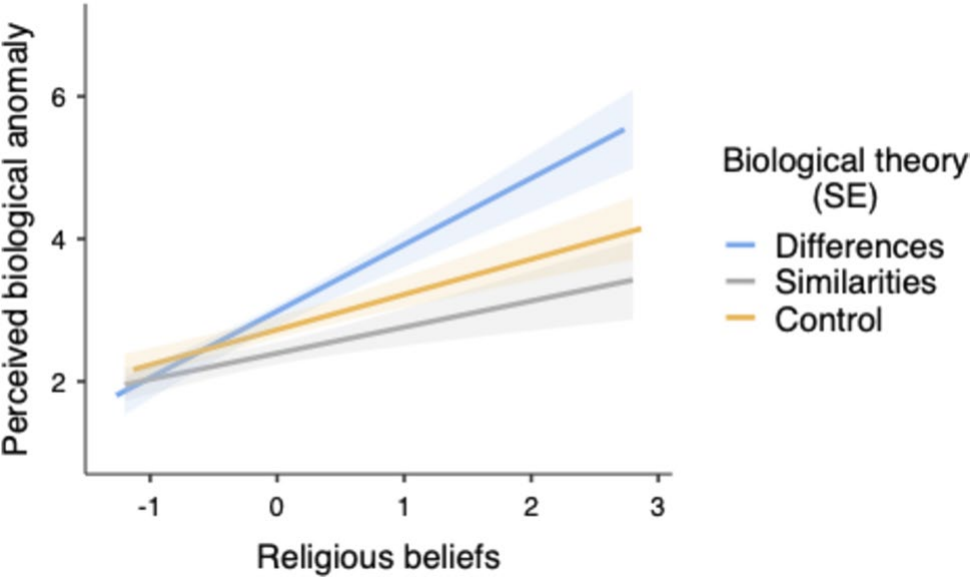
Predicted values for perception of homosexuality as a biological anomaly as a function of religiosity and biological theory framing (Study 2)

The interaction between religiosity and C1 was also significant,* F*(1,268) = 5.34, *p* = 0.021, η^2^_p_ = 0.020 (see Fig. 6). When religiosity was low (-1*SD*), the differences condition did not differ from the other two conditions (C1), *t*(268) = 0.57, *p* = 0.56, η^2^_p_ = 0.001, and those two conditions did not differ from each other (C2), *t*(268) = 0.81, *p* = 0.41, η^2^_p_ = 0.002. When religiosity was high (+ 1*SD*), the differences condition differed from the other two conditions (C1), *t*(268) = 2.81, *p* = 0.005, η^2^_p_ = 0.029, and those conditions did not differ from each other (C2), *t*(286) = 1.45, *p* > 0.14, η^2^_p_ = 0.008. Furthermore, religiosity was related to a higher perception of homosexuality as a biological anomaly in the three experimental conditions, but this effect was stronger in the differences condition (*B* = 0.93, *SE* = 0.18), *t*(268) = 5.03, *p* < 0.001, η^2^_p_ = 0.086, than in the similarities condition (*B* = 0.36, *SE* = 0.17), *t*(268) = 2.07, *p* = 0.039, η^2^_p_ = 0.016, and in the control condition (*B* = 0.49, *SE* = 0.14), *t*(268) = 3.46, *p* = 0.001, η^2^_p_ = 0.043.

Moreover, the interaction between participant’s sex and religiosity was also significant, *F*(1, 268) = 4.12, *p* < 0.043, η^2^_p_ = 0.015, indicating that the link between religiosity and perceived biological anomaly was stronger among male participants (*B* = 0.79, *SE* = 0.16), *t*(268) = 4.75, *p* < 0.001, η^2^_p_ = 0.078, than among female participants (*B* = 0.40, *SE* = 0.10), *t*(268) = 3.96, *p* < 0.001, η^2^_p_ = 0.055. Whereas the effect of sex was not significant when religiosity was low (-1*SD*), *t*(268) = 1.91, *p* = 0.056, η^2^_p_ = 0.014, men perceived homosexuality as a biological anomaly to a greater extent than women, *t*(268) = 4.52, *p* < 0.001, η^2^_p_ = 0.071. Finally, none of the interactions between participant’s sex and either C1 or C2 was significant,* F*s < 2.80, *p*s > 0.095, η^2^_p_ < 0.01.

**Fig. 7:**
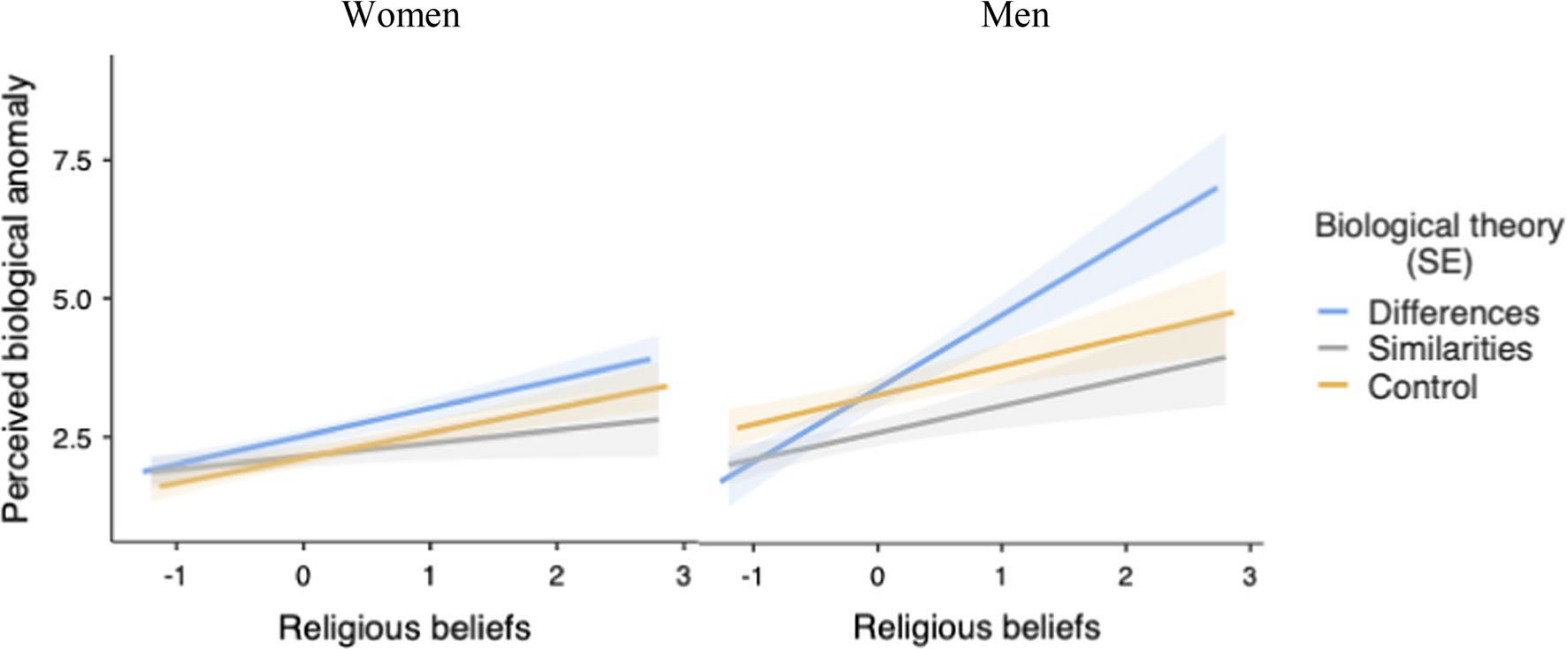
Predicted values for the perception of homosexuality as a biological anomaly as a function of religiosity, biological theory framing, and participant gender (Study 2)

Thus, the critical interaction between sex, religiosity and C1 was not significant, *F*(1, 268) = 2.45, *p* = 0.118, η^2^_p_ = 0.009. However, given that this analysis revealed two first-order interactions (religiosity × sex and religiosity × C1), and that participants’ sex moderated the predicted religiosity × C1 interaction for attitudes toward homosexuality, for exploratory purposes, we also examined this interaction separately for male and female participants (see Fig. 7). These analyses revealed that none of the interactions with C2 was significant. Moreover, the predicted religiosity × C1 interaction was significant for male participants, *F*(1, 268) = 4.73, *p* = 0.03, η^2^_p_ = 0.017, but not for female participants, *F*(1, 268) = 0.67, *p* = 0.41, η^2^_p_ = 0.003. Among male participants, none of the two contrasts was significant when religiosity was low (-1*SD*; C1: *t*(268) = 1.03, *p* = 0.30, η^2^_p_ = 0.004; C2: *t*(268) = 1.53, *p* = 0.12, η^2^_p_ = 0.009). However, the C1 was significant when religiosity was high (+ 1*SD*), *t*(268) = 2.24, *p* = 0.025, η^2^_p_ = 0.019, which was not the case for C2, *t*(268) = 1.35, *p* = 0.17, η^2^_p_ = 0.007.

#### Perceived Controllability

The main ANCOVA on perceived controllability showed a significant main effect of participant’s sex, *F*(1,268) = 11.79, *p* = 0.001, η^2^_p_ = 0.042. Male participants (*M* = 3.24, *SD* = 1.54) perceived more controllability than female participants (*M* = 2.59, *SD* = 1.59). The analysis also revealed a significant main effect of religiosity, *F*(1,268) = 7.94, *p* = 0.005, η^2^_p_ = 0.029. Overall, religiosity was associated with a higher perceived controllability (*B* = 0.31, *SE* = 0.11). However, and conversely to the results observed in Study 1, no other effects were significant (see Fig. 3, right side).

### Discussion

The results of this study corroborate those from Study 1 regarding attitudes toward homosexuality, the main dependent variable. Religiosity was consistently associated with less favorable attitudes, with this relationship being stronger in the biological differences condition compared to the other two experimental conditions. Notably, this pattern was specific to male participants, suggesting that heterosexual women may be less inclined to strategically interpret BTSO evidence. In other words, while religiosity was associated with less favorable attitudes among women, this relationship was not influenced by the experimental manipulation.

For perceptions of homosexuality, the findings also aligned with our predictions. Religiosity was associated with an increased perception of homosexuality as a biological anomaly, an effect amplified in the biological differences condition. Although the overall interaction with participant sex was not significant, exploratory analyses revealed that the predicted interaction was significant only among male participants.

Finaly, religiosity was positively associated with higher perceived controllability, consistent with previous research (Haslam & Levy, 2006; Whitehead, 2010). However, unlike in Study 1, no significant effect of the biological theory frame was observed. While the findings for perceived controllability were inconsistent across the two studies, they align with past research suggesting that exposure to BTSO evidence cannot be fully explained by attribution theory alone (Falomir-Pichastor & Mugny, 2009; Hegarty, 2002).

## General Discussion

This research investigated the impact of exposure to BTSO evidence on heterosexual men’s sexual prejudice and the moderating role of religiosity. We hypothesized that exposure to BTSO evidence would reduce prejudice among heterosexual men with low religiosity but not among those with high religiosity. Results from two studies supported this hypothesis.

A key strength of this research is the use of diverse measures for both religiosity and sexual prejudice. Study 1 used the positive ATH (Anderson et al., 2018) and negative ATLG (Herek, 1988) scales, while Study 2 introduced a novel measure of homosexuality as a biological disorder alongside the ATH scale. Religiosity was assessed via self-identification as believers in Study 1 and through the centrality of religiosity scale (Huber & Huber, 2012) in Study 2. Consistent findings across these measures provide strong evidence for the main hypothesis.

The gender-specific effects observed align with previous research. Heterosexual men display greater sexual prejudice, particularly toward gay men, as they are motivated to assert masculinity by rejecting those who deviate from traditional masculinity standards (Bosson et al., 2005; Falomir‐Pichastor & Hegarty, 2014; Falomir-Pichastor & Mugny, 2009; Herek, 1986; Iacoviello et al., 2020; Talley & Bettencourt, 2008; Vandello et al., 2008). Heterosexual men with low religiosity interpreted BTSO evidence positively, as legitimizing human sexual diversity, while those with high religiosity viewed it negatively, as evidence of biological deviance. In contrast, heterosexual women showed no evidence of such strategic reinterpretation, although their attitudes were still negatively associated with religiosity. These findings underscore the interplay between religiosity, gender, and motivated reasoning in shaping responses to BTSO evidence.

### Biological Explanations for Sexual Behavior

This research extends our understanding of the relationship between BTSO evidence and sexual prejudice. First, past research suggests that the BTSO is not straightforwardly related to prejudice (Falomir‐Pichastor & Hegarty, 2014; Falomir-Pichastor & Mugny, 2009; Haslam & Levy, 2006). Instead, individuals interpret BTSO evidence through the lens of not only their pre-existing attitudes (Boysen & Vogel, 2007) and ideologies (Ching & Chen, 2022), but also their distinctiveness motivations based on their gender identity and social norms (Falomir-Pichastor et al., 2017). This research adds to this body of work by showing that religiosity also influences whether BTSO evidence is interpreted as supporting natural diversity or as indicating a biological anomaly.

Second, religious individuals often construct anti-homosexuality narratives to counter the attributional effects of BTSO evidence (Thomas & Whitehead, 2015; Whitehead, 2010). This research extends these findings by providing experimental evidence of biased processing among religious heterosexual men, demonstrating that religiosity shapes BTSO interpretations beyond attribution theory. These results align with motivated reasoning theory (Kunda, 1990), showing how religiosity biases perceptions of biological causes, leading to divergent conclusions about homosexuality.

Third, this research aligns with studies that highlight the limitations of attribution theory in explaining the effects of BTSO evidence on attitudes toward homosexuality (Hegarty, 2002; Hegarty & Golden, 2008). The findings challenge attributional explanations, as evidence supporting the BTSO did not consistently reduce perceptions of control over sexual orientation. Moreover, the results do not strongly support the idea that BTSO evidence increases perceptions of discreteness or intergroup differences (see Supplementary Material). Future research should investigate the specific conditions under which BTSO evidence increases perceived entitativity and personal dissimilarity.

Fourth, this research contributes to broader discussions about the effects of biological explanations across domains. While previous studies have focused on neurodiversity (Park et al., 2018) and sexual minorities (Haider-Markel & Joslyn, 2008; Hewitt & Moore, 2002), the role of religiosity remains underexplored. Our findings emphasize the importance of religiosity in shaping responses to evidence about the etiology of sexual orientation and suggest that further research should investigate whether religiosity similarly influences interpretations of biological explanations for other stigmatized behaviors.

Finally, this research reinforces the well-documented link between religiosity and sexual prejudice (Etengoff & Lefevor, 2021; Herek et al., 2007). However, this relationship is complex. Allport characterized the religion-prejudice link as paradoxical (Allport, 1954), with religion promoting both prejudice and prosocial values, such as unconditional acceptance (Preston & Ritter, 2013; Vilaythong et al., 2010). Religiosity’s relationship with prejudice appears to depend on individual motivations (Burch-Brown & Baker, 2016). This research contributes to this nuanced understanding by showing that heterosexual men and women are differently motivated to interpret BTSO evidence. Masculinity drives heterosexual men to distance themselves from gay men, amplifying biased processing of BTSO evidence. While BTSO evidence can satisfy differentiation needs and reduce sexual prejudice (Falomir-Pichastor & Mugny, 2009; Iacoviello et al., 2020) or reinforce intergroup differences (Keller, 2005; Yzerbyt et al., 2001), our findings suggest that religiosity moderates these effects. For heterosexual men, religiosity motivates interpreting BTSO evidence as divine proof that heterosexuality is natural and homosexuality is a deviation. Further research should examine the mechanisms and conditions underlying this relationship between religiosity, gender and attitudes toward sexual minorities.

### Limitations and Future Research Directions

This research has several limitations that should be addressed in future studies. First, the data were collected in Switzerland (French-speaking regions) and France, where Christianity (primarily Catholicism and Protestantism) is dominant. This limits the generalizability of the findings to other cultural and religious contexts. Future research should test these hypotheses in contexts with varying levels of religiosity, social tolerance, and legal protections for sexual minorities (Anderson & Koc, 2015; Etengoff & Lefevor, 2021; Saroglou, 2019). Second, religiosity is linked to factors such as political ideologies (Etengoff & Lefevor, 2021; Van Der Toorn et al., 2017), RWA (Stefurak et al., 2010; Tsang & Rowatt, 2007), and perceived threats to masculinity (Janssen & Scheepers, 2019; Reese et al., 2013). Future studies should examine whether religiosity moderates the effect of BTSO evidence on sexual prejudice independently of these factors. Third, while religiosity was operationalized differently in each study, it was treated as a single construct. Religiosity encompasses multiple dimensions (Tasker, 2014), and future research should examine whether findings hold across dimensions such as religious practice, strength of faith, or intrinsic versus extrinsic motivations (Batson & Stocks, 2005).

This research also focused on biological explanations of sexual orientation. Although evidence for environmental causes is limited (Bailey et al., 2016), future research should investigate whether gender and religiosity similarly moderate responses to environmental explanations, such as social influence or individual preferences. Additionally, the manipulation assumed that BTSO evidence inherently suggests biological differences between heterosexual and gay individuals. However, the design did not clearly separate beliefs about biological similarity from beliefs about biological determinants. Future studies should disentangle these perceptions to better understand their distinct effects. Finally, the manipulation was sex-specific, exposing male participants to comparisons between heterosexual men and gay men, and female participants to comparisons between heterosexual women and lesbians. While this approach simplified the design, future research should examine whether the findings generalize to gender-neutral information or whether specific effects emerge from sex-specific comparisons.

### Conclusion

The debate over whether sexual orientation is biologically determined remains central to social discourse with significant political implications. Our findings reveal that the relationship between deterministic beliefs and sexual prejudice is more nuanced than previously understood. It is not the scientific evidence supporting biological or social explanations that drives prejudice, but rather the individual and contextual factors that shape its interpretation. Beliefs about the biological basis of sexual orientation can either increase or reduce prejudice, depending on these influences. In this context, religion and gender function as cultural tools that individuals may use to either promote or resist gender and sexual equality (Etengoff & Lefevor, 2021; Falomir‐Pichastor & Hegarty, 2014; Falomir-Pichastor & Mugny, 2009). By highlighting the importance of recognizing and valuing diverse expressions of sexuality, this research contributes to the broader discussion of how sexual orientation is understood—whether as biologically determined or socially constructed (Alipour, 2017).

## Supplementary Information

Below is the link to the electronic supplementary material.

Supplementary file1 (DOCX 87 KB)

## Data Availability

Materials and data are publicly available on OSF.

## Footnotes

1 At the end of both studies, participants were debriefed and thanked. During the debriefing, they were informed about the study’s objectives and methods, and the fact that no one single (environmental or biological) factor explains sexual orientation. Both studies complied with APA ethical guidelines and received approval from the ethical committee of the first author’s institution. All measures, manipulations, and data exclusions are fully reported in this manuscript or the Supplementary Material. All materials were in French, and data are publicly available on OSF https://osf.io/67c3u/?view_only=1dc4930c2467436f8edf13b614d5029c.

2 The main analysis, conducted without excluding participants who did not self-identify as heterosexual (total *N* = 150), revealed a significant interaction between religiosity and the experimental condition for both main dependent variables: positive attitudes toward homosexuality, *F*(1, 146) = 10.96, *p* < .001, η^2^_p_ = .07, and negative attitude toward lesbians and gay men, *F*(1, 146) = 19.03, *p* < .001, η^2^_p_ = .11. While the results were similar regardless of whether non-heterosexual participants were excluded, the small number of non-heterosexuals limits any conclusions specific to this group. Therefore, we chose to present the results for the heterosexual sample for theoretical and methodological reasons.

3 We also conducted the main analysis described in the results section without excluding participants who did not self-identify as heterosexual (*N* = 369). For positive attitudes toward homosexuality, the predicted interaction between religiosity, and C1 remained significant,* F*(1, 357) = 4.47, *p* = .035, η^2^_p_ = .012, as did the three-way interaction between C1, religiosity and participant sex,* F*(1, 357) = 4.08, *p* = .044, η^2^_p_ = .011. For perceived biological anomaly, the predicted interaction between religiosity, and C1 was significant,* F*(1, 357) = 6.32, *p* = .012, η^2^_p_ = .017. However, the three-way interaction between C1, religiosity and participant sex did not reach conventional significance,* F*(1, 357) = 3.63, *p* = .057, η^2^_p_ = .010, though it was closer to the significance level compared to the analysis excluding non-heterosexual participants (see results section). In sum, as in Study 1, the results were similar whether or not we excluded non-heterosexual participants. However, due to the small number of non-heterosexual male participants, no conclusions can be drawn specifically for this group. For theoretical and methodological reasons, we chose to report the results excluding non-heterosexual participants.

## References

[CR1] Alipour, M. (2017). Essentialism and islamic theology of homosexuality: A critical reflection on an essentialist epistemology toward same-sex desires and acts in Islam. *Journal of Homosexuality,**64*(14), 1930–1942. 10.1080/00918369.2017.1289001 28139174 10.1080/00918369.2017.1289001

[CR2] Anderson, J. R., Ashford, L. J., Prakash, P., & Gerace, A. (2022). The role of religion in explaining the relationship between sexual prejudice and the rejection of marriage equality. *Psychology and Sexuality,**13*(3), 610–627. 10.1080/19419899.2021.1900346

[CR3] Anderson, J. R., & Koc, Y. (2015). Exploring patterns of explicit and implicit anti-gay attitudes in Muslims and atheists. *European Journal of Social Psychology,**45*(6), 687–701. 10.1002/ejsp.2126

[CR4] Anderson, J. R., Koc, Y., & Falomir-Pichastor, J. M. (2018). The English version of the Attitudes toward Homosexuality Scale. *Swiss Journal of Psychology,**77*(3), 117–126. 10.1024/1421-0185/a000210

[CR5] Bailey, J. M., Vasey, P. L., Diamond, L. M., Breedlove, S. M., Vilain, E., & Epprecht, M. (2016). Sexual orientation, controversy, and science. *Psychological Science in the Public Interest,**17*(2), 45–101. 10.1177/1529100616637616 27113562 10.1177/1529100616637616

[CR6] Batson, C. D., & Stocks, E. L. (2005). Religion and prejudice. In J. F. Dovidio, P. Glick, & L. A. Rudman (Eds.), *On the nature of prejudice* (pp. 413–427). Wiley. 10.1002/9780470773963.ch25

[CR7] Bosson, J. K., & Michniewicz, K. S. (2013). Gender dichotomization at the level of ingroup identity: What it is, and why men use it more than women. *Journal of Personality and Social Psychology,**105*(3), 425–442. 10.1037/a0033126 23750813 10.1037/a0033126

[CR8] Bosson, J. K., Prewitt-Freilino, J. L., & Taylor, J. N. (2005). Role rigidity: A problem of identity misclassification? *Journal of Personality and Social Psychology,**89*(4), 552–565. 10.1037/0022-3514.89.4.552 16287418 10.1037/0022-3514.89.4.552

[CR9] Boysen, G. A., & Vogel, D. L. (2007). Biased assimilation and attitude polarization in response to learning about biological explanations of homosexuality. *Sex Roles,**57*(9–10), 755–762. 10.1007/s11199-007-9256-7

[CR10] Burch-Brown, J., & Baker, W. (2016). Religion and reducing prejudice. *Group Processes & Intergroup Relations,**19*(6), 784–807. 10.1177/1368430216629566

[CR11] Carnaghi, A., Maass, A., & Fasoli, F. (2011). Enhancing masculinity by slandering homosexuals: The role of homophobic epithets in heterosexual gender identity. *Personality and Social Psychology Bulletin,**37*(12), 1655–1665. 10.1177/0146167211424167 21975948 10.1177/0146167211424167

[CR12] Ching, B. H.-H., & Chen, T. T. (2022). Effects of biological determinism on beliefs and attitudes about transgender people: Psychological essentialism and biased assimilation. *Archives of Sexual Behavior,**51*(4), 1927–1942. 10.1007/s10508-021-02262-8 35459970 10.1007/s10508-021-02262-8

[CR13] Ching, B.H.-H., Xu, J. T., Chen, T. T., & Kong, K. H. C. (2020). Gender essentialism, authoritarianism, social dominance orientation, and filial piety as predictors for transprejudice in Chinese people. *Sex Roles,**83*(7–8), 426–441. 10.1007/s11199-020-01123-3

[CR14] Cook, C. C. H. (2021). The causes of human sexual orientation. *Theology & Sexuality,**27*(1), 1–19. 10.1080/13558358.2020.1818541

[CR15] Drescher, J. (2015). Out of DSM: Depathologizing homosexuality. *Behavioral Sciences,**5*(4), 565–575. 10.3390/bs5040565 26690228 10.3390/bs5040565 PMC4695779

[CR150] Ernulf, K. E, Innala, S. M., & Whitam, F. L. (1989). Biological explanation, psychological explanation, and tolerance of homosexuals: A cross-national analysis of beliefs and attitudes. *Psychological Reports*, *65*(3), 1003–1010. 10.2466/pr0.1989.65.3.1003 2608821 10.2466/pr0.1989.65.3.1003

[CR16] Etengoff, C., & Lefevor, T. G. (2021). Sexual prejudice, sexism, and religion. *Current Opinion in Psychology,**40*, 45–50. 10.1016/j.copsyc.2020.08.024 33007574 10.1016/j.copsyc.2020.08.024

[CR17] Falomir-Pichastor, J. M., & Hegarty, P. (2014). Maintaining distinctions under threat: Heterosexual men endorse the biological theory of sexuality when equality is the norm. *British Journal of Social Psychology,**53*(4), 731–751. 10.1111/bjso.12051 24131397 10.1111/bjso.12051

[CR18] Falomir-Pichastor, J. M., & Mugny, G. (2009). “I’m not gay…I’m a real man!”: Heterosexual men’s gender self-esteem and sexual prejudice. *Personality and Social Psychology Bulletin,**35*(9), 1233–1243. 10.1177/0146167209338072 19571277 10.1177/0146167209338072

[CR19] Falomir-Pichastor, J. M., Mugny, G., & Berent, J. (2017). The side effect of egalitarian norms: Reactive group distinctiveness, biological essentialism, and sexual prejudice. *Group Processes & Intergroup Relations,**20*(4), 540–558. 10.1177/1368430215613843

[CR20] Finlay, B., & Walther, C. S. (2003). The relation of religious affiliation, service attendance, and other factors to homophobic attitudes among university students. *Review of Religious Research,**44*(4), 370. 10.2307/3512216

[CR21] Frias-Navarro, D., Monterde-i-Bort, H., Pascual-Soler, M., & Badenes-Ribera, L. (2015). Etiology of homosexuality and attitudes toward same-sex parenting: A randomized study. *Journal of Sex Research,**52*(2), 151–161. 10.1080/00224499.2013.802757 24024528 10.1080/00224499.2013.802757

[CR22] Haider-Markel, D. P., & Joslyn, M. R. (2008). Beliefs about the origins of homosexuality and support for gay rights: An empirical test of attribution theory. *Public Opinion Quarterly,**72*(2), 291–310. 10.1093/poq/nfn015

[CR23] Halley, J. E. (1994). Sexual orientation and the politics of biology: A critique of the argument from immutability. *Stanford Law Review,**46*(3), 503. 10.2307/1229101

[CR24] Haslam, N., & Levy, S. R. (2006). Essentialist beliefs about homosexuality: Structure and implications for prejudice. *Personality and Social Psychology Bulletin,**32*(4), 471–485. 10.1177/0146167205276516 16513800 10.1177/0146167205276516

[CR25] Haslam, N., Rothschild, L., & Ernst, D. (2002). Are essentialist beliefs associated with prejudice? *British Journal of Social Psychology,**41*(1), 87–100. 10.1348/014466602165072 11970776 10.1348/014466602165072

[CR26] Hegarty, P. (2002). ‘It’s not a choice, it’s the way we’re built’: Symbolic beliefs about sexual orientation in the US and Britain. *Journal of Community & Applied Social Psychology,**12*(3), 153–166. 10.1002/casp.669

[CR27] Hegarty, P. (2010). A stone in the soup? Changes in sexual prejudice and essentialist beliefs among British students in a class on LGBT psychology. *Psychology and Sexuality,**1*(1), 3–20. 10.1080/19419891003634356

[CR28] Hegarty, P., & Golden, A. M. (2008). Attributional beliefs about the controllability of stigmatized traits: Antecedents or justifications of prejudice? *Journal of Applied Social Psychology,**38*(4), 1023–1044. 10.1111/j.1559-1816.2008.00337.x

[CR29] Herek, G. M. (1986). On heterosexual masculinity: Some psychical consequences of the social construction of gender and sexuality. *American Behavioral Scientist,**29*(5), 563–577. 10.1177/000276486029005005

[CR30] Herek, G. M. (1987). Religious orientation and prejudice: A comparison of racial and sexual attitudes. *Personality and Social Psychology Bulletin,**13*(1), 34–44. 10.1177/0146167287131003

[CR31] Herek, G. M. (1988). Heterosexuals’ attitudes toward lesbians and gay men: Correlates and gender differences. *Journal of Sex Research,**25*(4), 451–477. 10.1080/00224498809551476

[CR32] Herek, G. M., Chopp, R., & Strohl, D. (2007). Sexual stigma: Putting sexual minority health issues in context. In I. H. Meyer & M. E. Northridge (Eds.), *The health of sexual minorities: Public health perspectives on lesbian, gay, bisexual, and transgender populations* (pp. 171–208). Springer.

[CR33] Herek, G. M., & McLemore, K. A. (2013). Sexual prejudice. *Annual Review of Psychology,**64*(1), 309–333. 10.1146/annurev-psych-113011-143826 22994920 10.1146/annurev-psych-113011-143826

[CR34] Hewitt, E. C., & Moore, L. D. (2002). The role of lay theories of the etiologies of homosexuality in attitudes toward lesbians and gay men. *Journal of Lesbian Studies,**6*(3–4), 58–72. 10.1300/J155v06n03_06 24804588 10.1300/J155v06n03_06

[CR35] Holdcroft, B. B. (2006). What is religiosity. *Journal of Catholic Education*, *10*(1). 10.15365/joce.1001082013

[CR36] Huber, S., & Huber, O. W. (2012). The Centrality of Religiosity Scale (CRS). *Religions,**3*(3), 710–724. 10.3390/rel3030710

[CR37] Iacoviello, V., Valsecchi, G., Berent, J., Anderson, J., & Falomir-Pichastor, J. M. (2020). Heterosexual men’s attitudes toward homosexuality and ingroup distinctiveness: The role of perceived men’s feminisation. *Psychology & Sexuality,**11*(1–2), 45–61. 10.1080/19419899.2019.1675749

[CR38] Janssen, D.-J., & Scheepers, P. (2019). How religiosity shapes rejection of homosexuality across the globe. *Journal of Homosexuality,**66*(14), 1974–2001. 10.1080/00918369.2018.1522809 30372378 10.1080/00918369.2018.1522809

[CR39] Jayaratne, T. E., Ybarra, O., Sheldon, J. P., Brown, T. N., Feldbaum, M., Pfeffer, C. A., & Petty, E. M. (2006). White Americans’ genetic lay theories of race differences and sexual orientation: Their relationship with prejudice toward blacks, and gay men and lesbians. *Group Processes & Intergroup Relations,**9*(1), 77–94. 10.1177/1368430206059863 10.1177/1368430206059863 PMC3832063 24260013

[CR40] Jonathan, E. (2008). The influence of religious fundamentalism, right-wing authoritarianism, and Christian orthodoxy on explicit and implicit measures of attitudes toward homosexuals. *International Journal for the Psychology of Religion,**18*(4), 316–329. 10.1080/10508610802229262

[CR41] Keller, J. (2005). In genes we trust: The biological component of psychological essentialism and its relationship to mechanisms of motivated social cognition. *Journal of Personality and Social Psychology,**88*(4), 686–702. 10.1037/0022-3514.88.4.686 15796668 10.1037/0022-3514.88.4.686

[CR42] Khan, S. S., Tarrant, M., Weston, D., Shah, P., & Farrow, C. (2018). Can raising awareness about the psychological causes of obesity reduce obesity stigma? *Health Communication,**33*(5), 585–592. 10.1080/10410236.2017.1283566 28278610 10.1080/10410236.2017.1283566

[CR43] Kite, M. E., Whitley, B. E., Buxton, K., & Ballas, H. (2021). Gender differences in anti-gay prejudice: Evidence for stability and change. *Sex Roles,**85*(11–12), 721–750. 10.1007/s11199-021-01227-4

[CR44] Kunda, Z. (1990). The case for motivated reasoning. *Psychological Bulletin,**108*(3), 480–498. 10.1037/0033-2909.108.3.480 2270237 10.1037/0033-2909.108.3.480

[CR45] Kvaale, E. P., Haslam, N., & Gottdiener, W. H. (2013). The ‘side effects’ of medicalization: A meta-analytic review of how biogenetic explanations affect stigma. *Clinical Psychology Review,**33*(6), 782–794. 10.1016/j.cpr.2013.06.002 23831861 10.1016/j.cpr.2013.06.002

[CR46] Layman, G. C., & Carmines, E. G. (1997). Cultural conflict in American politics: Religious traditionalism, postmaterialism, and US political behavior. *Journal of Politics,**59*(3), 751–777. 10.2307/2998636

[CR47] Lord, C. G., Ross, L., & Lepper, M. R. (1979). Biased assimilation and attitude polarization: The effects of prior theories on subsequently considered evidence. *Journal of Personality and Social Psychology,**37*(11), 2098–2109. 10.1037/0022-3514.37.11.2098

[CR48] Moore, D. A., Nunns, M., Shaw, L., Rogers, M., Walker, E., Ford, T., Garside, R., Ukoumunne, O., Titman, P., Shafran, R., Heyman, I., Anderson, R., Dickens, C., Viner, R., Bennett, S., Logan, S., Lockhart, F., & Thompson Coon, J. (2019). Interventions to improve the mental health of children and young people with long-term physical conditions: Linked evidence syntheses. *Health Technology Assessment,**23*(22), 1–164. 10.3310/hta23220 31122334 10.3310/hta23220 PMC6556821

[CR49] Morton, T. A., & Postmes, T. (2009). When differences become essential: Minority essentialism in response to majority treatment. *Personality and Social Psychology Bulletin,**35*(5), 656–668. 10.1177/0146167208331254 19228599 10.1177/0146167208331254

[CR50] Oldham, J., & Kasser, T. (1999). Attitude change in response to information that male homosexuality has a biological basis. *Journal of Sex & Marital Therapy,**25*(2), 121–124. 10.1080/00926239908403984 10327380 10.1080/00926239908403984

[CR51] Park, S., Lee, Y., & Kim, C. E. (2018). Korean adults’ beliefs about and social distance toward attention-deficit hyperactivity disorder, Tourette syndrome, and autism spectrum disorder. *Psychiatry Research,**269*, 633–639. 10.1016/j.psychres.2018.08.023 30212793 10.1016/j.psychres.2018.08.023

[CR52] Phelan, J. C. (2005). Geneticization of deviant behavior and consequences for stigma: The case of mental illness. *Journal of Health and Social Behavior,**46*(4), 307–322. 10.1177/002214650504600401 16433278 10.1177/002214650504600401

[CR53] Piskur, J., & Degelman, D. (1992). Effect of reading a summary of research about biological bases of homosexual orientation on attitudes toward homosexuals. *Psychological Reports,**71*(3), 1219–1225. 10.2466/pr0.1992.71.3f.1219 1480708 10.2466/pr0.1992.71.3f.1219

[CR54] Pratarelli, M. E., & Donaldson, J. S. (1997). Immediate effects of written material on attitudes toward homosexuality. *Psychological Reports,**81*(3), 1411–1415. 10.2466/pr0.1997.81.3f.1411 9461776 10.2466/pr0.1997.81.3f.1411

[CR55] Preston, J. L., & Ritter, R. S. (2013). Different effects of religion and god on prosociality with the ingroup and outgroup. *Personality and Social Psychology Bulletin,**39*(11), 1471–1483. 10.1177/0146167213499937 23969621 10.1177/0146167213499937

[CR56] Reese, G., Steffens, M. C., & Jonas, K. J. (2013). When black sheep make us think: Information processing and devaluation of in- and outgroup norm deviants. *Social Cognition,**31*(4), 482–503. 10.1521/soco_2012_1005

[CR57] Saroglou, V. (2019). Religion and related morality across cultures. In V. Saroglou (Ed.), *The handbook of culture and psychology* (pp. 724–785). Oxford University Press. 10.1093/oso/9780190679743.003.0022

[CR58] Shostak, S., Freese, J., Link, B. G., & Phelan, J. C. (2009). The politics of the gene: Social status and beliefs about genetics for individual outcomes. *Social Psychology Quarterly,**72*(1), 77–93. 10.1177/019027250907200107 25400308 10.1177/019027250907200107 PMC4228480

[CR59] Sloane, J. L., & Robillard, L. M. (2018). Factors affecting heterosexual attitudes to same-sex marriage in Australia. *Sexuality Research and Social Policy,**15*(3), 290–301. 10.1007/s13178-017-0276-y

[CR60] Stefurak, T., Taylor, C., & Mehta, S. (2010). Gender-specific models of homosexual prejudice: Religiosity, authoritarianism, and gender roles. *Psychology of Religion and Spirituality,**2*(4), 247–261. 10.1037/a0021538

[CR61] Talley, A. E., & Bettencourt, B. A. (2008). Evaluations and aggression directed at a gay male target: The role of threat and antigay prejudice. *Journal of Applied Social Psychology,**38*(3), 647–683. 10.1111/j.1559-1816.2007.00321.x

[CR62] Tasker, T. B. (2014). *Multi-dimensionality and the complex relationships between religious belief and sexual prejudice*. University of Illinois at Chicago. https://hdl.handle.net/10027/11244

[CR63] Thomas, J. N., & Olson, D. V. A. (2012). Evangelical elites’ changing responses to homosexuality 1960–2009. *Sociology of Religion,**73*(3), 239–272. 10.1093/socrel/srs031

[CR64] Thomas, J. N., & Whitehead, A. L. (2015). Evangelical elites’ anti-homosexuality narratives as a resistance strategy against attribution effects. *Journal for the Scientific Study of Religion,**54*(2), 345–362. 10.1111/jssr.12188

[CR65] Tsang, J.-A., & Rowatt, W. C. (2007). The relationship between religious orientation, right-wing authoritarianism, and implicit sexual prejudice. *International Journal for the Psychology of Religion,**17*(2), 99–120. 10.1080/10508610701244122

[CR66] Tygart, C. E. (2000). Genetic causation attribution and public support of gay rights. *International Journal of Public Opinion Research,**12*(3), 259–275. 10.1093/ijpor/12.3.259

[CR67] Van Der Toorn, J., Jost, J. T., Packer, D. J., Noorbaloochi, S., & Van Bavel, J. J. (2017). In Defense of tradition: Religiosity, conservatism, and opposition to same-sex marriage in North America. *Personality and Social Psychology Bulletin,**43*(10), 1455–1468. 10.1177/0146167217718523 28918711 10.1177/0146167217718523 PMC5665159

[CR68] Vandello, J. A., Bosson, J. K., Cohen, D., Burnaford, R. M., & Weaver, J. R. (2008). Precarious manhood. *Journal of Personality and Social Psychology,**95*(6), 1325–1339. 10.1037/a0012453 19025286 10.1037/a0012453

[CR69] Verkuyten, M. (2003). Discourses about ethnic group (de-)essentialism: Oppressive and progressive aspects. *British Journal of Social Psychology,**42*(3), 371–391. 10.1348/014466603322438215 14567843 10.1348/014466603322438215

[CR70] Vilaythong, T. O., Lindner, N. M., & Nosek, B. A. (2010). “Do unto others”: Effects of priming the golden rule on Buddhists’ and Christians’ attitudes toward gay people. *Journal for the Scientific Study of Religion,**49*(3), 494–506. 10.1111/j.1468-5906.2010.01524.x

[CR71] Weiner, B. (1993). On sin versus sickness: A theory of perceived responsibility and social motivation. *American Psychologist,**48*(9), 957–965. 10.1037/0003-066X.48.9.957 8214914 10.1037//0003-066x.48.9.957

[CR72] Whitehead, A. L. (2010). Sacred rites and civil rights: religion’s effect on attitudes toward same-sex unions and the perceived cause of homosexuality. *Social Science Quarterly,**91*(1), 63–79. 10.1111/j.1540-6237.2010.00681.x

[CR73] Whitley, B. E. (1990). The relationship of heterosexuals’ attributions for the causes of homosexuality to attitudes toward lesbians and gay men. *Personality and Social Psychology Bulletin,**16*(2), 369–377. 10.1177/0146167290162016

[CR74] Whitley, B. E. (2009). Religiosity and attitudes toward lesbians and gay men: A meta-analysis. *International Journal for the Psychology of Religion,**19*(1), 21–38. 10.1080/10508610802471104

[CR75] Whitley, B. E., Jr., & Ægisdóttir, S. (2000). The gender belief system, authoritarianism, social dominance orientation, and heterosexuals’ attitudes toward lesbians and gay men. *Sex Roles,**42*(11/12), 947–967. 10.1023/A:1007026016001

[CR76] Yzerbyt, V., Corneille, O., & Estrada, C. (2001). The interplay of subjective essentialism and entitativity in the formation of stereotypes. *Personality and Social Psychology Review,**5*(2), 141–155. 10.1207/S15327957PSPR0502_5

