# Supplementary material for: Perceived Biological Bases of Sexual Orientation and Sexual Prejudice: The Moderating Role of Gender and Religious Beliefs
Source: Arch Sex Behav. 2024 Dec 23;54(3):1245–59. doi: 10.1007/s10508-024-03070-6 (PMC11926014; doi:10.1007/s10508-024-03070-6)
Supplement: Supplementary file 1 — Supplementary file1 (DOCX 87 KB) [file 10508_2024_3070_MOESM1_ESM.docx]

**SUPPLEMENTARY MATERIAL**

1. **Pilot study**
2. **Additional materials**

**1) PILOT STUDY**

In order to provide a first test of our hypothesis, we ran a pilot study in which, instead of assessing participants’ religiosity, we rather focused on participants’ religious affiliation. We recruited a sample of both Atheistic and Catholic male participants in the realm of two master dissertations.^[[1]](#footnote-1)^ Specifically, we assessed individuals’ self-declaration as either members of a religious community (i.e., Catholics) or as Atheists, we experimentally manipulated the biological bases of sexual orientation as in Study 1 (main document), and assessed the main dependent variable (attitude towards homosexuality) as in Studies 1 and 2. As in the two main studies described in the main document, this pilot study also assessed participants’ perception of intergroup differences between heterosexual and gay men.

Results showed that, in the biological differences condition, Catholic male participants showed more negative attitudes towards homosexuality as compared to Atheist male participants, but this effect was not significant in the biological similarities condition. Furthermore, this pattern was similar for the additional dependent variable (perceived intergroup differences. Therefore, these findings provide evidence in support of the main hypothesis investigated in this research, this time whilst focusing on participants’ religious affiliation rather than religiosity.

Despite the relevance of these results, this pilot study is not described in the main document for simplicity and length reasons, but also because our main hypothesis focuses on religiosity (rather than religious affiliation), the sample combined two sub-samples that were recruited within two different master dissertations, and the sample size was nevertheless relatively small for a 2 x 2 experimental design. However, for transparency purposes, we describe method and results of this pilot study here in this Supplementary Material.

**Method**

**Participants and procedure**. This research was conducted as a part of two different master theses. Whereas each thesis had specific research goals and materials, the two shared the materials described in the present pilot study.^[[2]](#footnote-2)^ Participants were recruited to volunteer in an online questionnaire about sexual orientation and sexual prejudice. From the 347 participants initially recruited, 61 were excluded because they did not identify as heterosexual (see Materials: Sexual orientation). For the analyses, we retained self-declared Catholics (*n* = 79) and atheists (*n* = 101). The next largest groups were Protestants (*n* = 44), agnostics (*n* = 24), and Muslims (*n* = 24), but their number was too small to allow an appropriate test of our hypothesis. The final sample thus comprised 180 heterosexual people (80 students and 47 females; *M*_age_ = 21.66 years, *SD* = 3.70). They were randomly assigned to one of the two conditions of a 2 (*biological theory*: differences vs. similarities) between-participants design. A sensitivity power analysis on G*Power for an ANOVA with four groups (α = .05, two-tailed, and a power of .80) revealed that our final sample was powered enough to detect a small to medium effect size (*f* = 0.20). All materials were in French. Participants were randomly attributed to one of the two experimental conditions (*BTSO*: biological differences vs. biological similarities).

**Materials.**

***Biological theory of sexual orientation (BTSO)*.** The biological theory was manipulated as in Study 1 (Falomir-Pichastor & Mugny, 2009, Study 5). The material was gender-specific – that is, women and men received information based on their own gender. Participants read a text that summarized scientific evidence comparing heterosexual and gay men (or heterosexual and lesbian women, as a function of participant gender) with respect to their genes, mother’s androgen rate during pregnancy, and neurological make-up (i.e., the weight of the part of the hypothalamus responsible for sexual orientation). In the *biological differences condition*, the results of these studies highlighted the existence of biological differences between heterosexual and gay men (or heterosexual and lesbian women), thereby suggesting that sexual orientation is determined biologically. In the *biological similarities condition*, the results emphasized that heterosexual and gay men (or heterosexual and lesbian women) are biologically similar, indicating the lack of scientific evidence for biological determinism of sexual orientation.

***Manipulation checks***. We tested the manipulation of the biological theory through a single item: “Homosexuality is biologically determined” (1 ‘*not at all*’ to 7 ‘*absolutely*’; *M* = 3.05, *SD* = 1.82).

***Positive attitude towards homosexuality.*** We used a 16-item scale measuring participants' attitude towards homosexuality (e.g., ‘Gay couples should have the right to marry’; 1 = ‘*strongly disagree’* to 7 = ‘*strongly agree’*; Anderson et al., 2019). An overall score measuring positive attitudes was computed by averaging the answers to all items after reverse-coding appropriate items (*M* = 5.02, *SD* = 1.13, α = .90).

***Perceived intergroup differences***. We measured perceived entitativity through 3 items: “One can easily identify gay people by their way of being and behaving”, “Gay people and heterosexuals have different emotional characteristics”, and “Gay people and heterosexuals have different personality traits” (1 ‘*strongly disagree*’ to 7 ‘*strongly agree’*). Scores for these items were averaged to form a reliable measure of intergroup differentiation (higher scores indicate greater differentiation; *M* = 3.63, *SD* = 1.49, α = .82).

***Perceived controllability***. We measured perceived controllability through 3 items: “A gay man, at some point in his life, decides his sexual orientation voluntarily”, “Gay people have the possibility to change his sexual orientation”, and “Gay people are personally responsible for their sexual orientation” (1 ‘*strongly disagree*’ to 7 ‘*strongly agree*’). Scores for these items were averaged to form a reliable measure of perceived controllability (higher scores indicate greater perceived controllability; *M* = 3.22, *SD* = 1.67, α = .81).

***Sexual orientation*.** At the end of the study, participants responded to several demographic items including three questions about their sexual orientation: they defined themselves as ‘heterosexual’, ‘bisexual’, or ‘homosexual’, indicated previous sexual relations with a person of the same-sex (‘yes’ vs. ‘no’), and indicated whether they felt attracted to people of the same-sex (1 ‘*never*’ to 7 ‘*frequently*’). If participants defined themselves as heterosexual, reported not having had sexual relationships with a same-sex person, and scored below the mid-point of the attraction item scale (4), then they were categorized as heterosexual and retained for the analyses (Falomir‐Pichastor & Hegarty, 2014).

**Results**

Dependent variables were analyzed using a 2 (*religious affiliation*: Catholic, Atheist) x 2 (*biological theory*: differences, similarities) ANOVA.^[[3]](#footnote-3)^

**Manipulation check*.*** Regarding the endorsement of the biological theory, results revealed a main effect of the biological theory manipulation, *F*(1,175) = 17.39, *p* < .001, η_p_^2^ = .09. Participants endorsed more strongly that sexual orientation is biologically determined following the biological-differences manipulation (*M* = 3.59, *SD* = 1.66) compared to the biological-similarities manipulation (*M* = 2.45, *SD* = 1.82). No other main or interaction effects approached significance, *F*s < 0.48, *p*s > .48.

**Positive attitude towards homosexuality**. Regarding the main dependent variable, the ANOVA revealed a main effect of religious beliefs, *F*(1,176) = 10.81, *p* < .001, η_p_^2^ = .058. Atheists showed more positive attitudes towards homosexuality (*M* = 5.27, *SD* = 1.11) than Catholics (*M* = 4.70, *SD* = 1.07). Moreover, the predicted two-way interaction was also significant, *F*(1,176) = 5.49, *p* = .02, η_p_^2^ = .03 (see Figure 1). Among Catholics, the effect of the biological theory was not significant, *p* > .27, 95% CI [-0.75, 0.21], but Atheists showed more positive attitudes towards homosexuality in the biological differences condition than in the biological similarities condition, *p* < .023, 95% CI [0.06, 0.93]. We also decomposed the interaction the other way around. In the biological differences condition attitude towards homosexuality were less positive among Catholics (*M* = 4.55, *SD* = 1.23) than among Atheists (*M* = 5.47, *SD* = 0.99), *p* < .001, 95% *IC* [-1.38, -0.47]. However, this effect was not significant in the biological similarities condition (*M* = 4.81, *SD* = 0.93, and *M* = 4.97, *SD* = 1.21, respectively), *p* > .50, 95% CI [-0.61, 0.30].

**Figure 1.** Positive attitude towards homosexuality and perceived group entitativity as a function of participants’ religiosity and biological theory condition (bars represent ±1 standard deviation).

**Perceived intergroup differences**. The ANOVA only revealed a significant interaction effect on perceived intergroup differences, *F*(1,176) = 10.07, *p* = .002, η_p_^2^ = .054 (see Figure 1). Among Atheists, perceived differences were lower in the biological differences condition than in the biological similarities condition, *p* = .008, 95% CI [-1.37, -0.20]. However, the reverse tended to approach significance among Catholics, *p* = .064, 95% CI [-1.26, 0.03]. In the biological differences condition, Atheists (*M* = 3.20, *SD* = 1.26) showed less entitativity than Catholics (*M* = 4.11, *SD* = 1.51), *p* = .004, 95% CI [-1.52, -0.30]. However, this difference was not significant in the biological similarities condition (*M* = 3.99, *SD* = 1.67, and *M* = 3.50, *SD* = 1.44, respectively), *p* > .12, 95% CI [-0.13, 1.11].

**Perceived Controllability**. The ANOVA did not reveal any significant interaction effect, *F*s < 1.34, *ps* > .24.

**Discussion**

The results of this pilot study showed that religious affiliation, as assessed through the difference between Catholic and Atheist participants, increased sexual prejudice to a greater extent when science was thought to support the biological bases of sexual orientation. This pattern was also observed regarding perceived intergroup differences. As compared to Atheists, Catholics perceived gay men to a greater extent as a different group when exposed to scientific evidence in support of the biological bases of sexual orientation, but not when this evidence supported the lack of a biological determinism. In sum, the results of this pilot study are consistent with those observed in Studies 1 and 2, and therefore provide evidence in support of the main hypothesis.

**2) ADDITIONAL MATERIAL**

As already stated in the main document, the studies described were conducted independently by two master students as a part of their master dissertation, and these studies had specific goals that did not completely suit the main goal of the present research. Therefore, for both studies the questionnaires included specific materials that are beyond the scope and length of the main document. We decided not to present these measures in the main document because of their exploratory nature, their non-systematic inclusion across the two studies, and their inconsistent results, and for length and concision purposes. However, for transparency purposes we describe below all these additional materials.

**STUDY 1**

**Independent variables**. Beyond the material described in the main document, the questionnaire for Study 1 also included at pre-test a measure of endorsement of traditional masculinity norms (Thompson & Pleck, 1986) and a self-made experimental manipulation of the gender prototypicity of gay men (i.e., whether personality tests show that gay men are feminine as heterosexual women or masculine as heterosexual men).

However, these two additional independent variables were related to specific research goals of the student’s master thesis and were not included in Study 2. Moreover, the analyses including the four independent variables, as well as their interactions, revealed that the critical religiosity by BTSO interaction effect was significant and any of the two additional independent variables moderated this effect. Nevertheless, the small final sample in Study 1 (*N* = 118 participants) did not provide the sufficient power to test a 4-way interaction resulting from a complex design including four predictors: two individual differences measures (religiosity and masculinity norm endorsement) and two experimental manipulations (BTSO and gay gender prototypicity). Finally, it’s worth noting that these two additional independent variables are beyond the theoretical scope of the present manuscript which specifically focuses on the interplay between religiosity, gender and BTSO. Therefore, for simplicity and length concerns, in the main document we decided to focus on the religiosity by BTSO interaction investigated in the present research, and decided not to report the other two additional independent variables. More information about the specific goals, materials and results regarding these two additional independent variables can be obtained from the first author upon request.

**Dependent variables**. In Study 1, the questionnaire also included three additional scales assessing psychological differentiation for exploratory purposes. We describe below these measures and the results.

*Perceived intergroup differences (PIGD)*. We included a 3-item scale to measure participants’ perception of intergroup differences between heterosexual and gay individuals: “One can easily identify gay people by their way of being and behaving”, “Gay people and heterosexuals have different emotional characteristics”, and “Gay people and heterosexuals have different personality traits” (1 ‘*strongly disagree*’ to 7 ‘*strongly agree’*). The scores for these items were averaged to create a reliable measure of intergroup differentiation, with higher scores indicating greater differentiation (*M* = 3.89, *SD* = 1.79, α = .87).

*Traditional beliefs about gender and gender identity (TBG&GI).* We also included 15-item scale to assess traditional beliefs about gender and gender identity (Dasgupta & Rivera, 2006). This scale consists of two subscales: one measuring *traditional beliefs about gender* subscale assesses the degree to which people endorse traditional gender norms in various life domains (e.g., parenting, professional life, social interactions, or physical appearance; e.g., ‘It is important that men appear masculine and that women appear feminine’; *M* = 3.32, *SD* = 1.38, α = .84), and another measuring *traditional beliefs about gender identity* emphasizing heterosexual identity (e.g., ‘If a member of my sex made a sexual advance toward me I would feel angry’; *M* = 3.75, *SD* = 1.24, α = .73). Note that the second subscale assesses participants’ affirmation of (hetero)sexual identity, making it relevant to examine personal differentiation dynamics. However, both subscales were moderately correlated, *r*(118) = .37, *p* < .001, and we computed an overall score of TBG&GI (*M* = 3.55, *SD* = 1.08, α = .82).

*Perceived interpersonal similarity (PIPS)*. Finally, we included a 6-item scale to measure participants’ perceived similarity between themselves and gay men across various areas. We asked participants to rate their similarity at the biological and psychological levels, emotional level, way of functioning, way of relating, and in general (1 ‘*strongly different*’ to 7 ‘*strongly similar’*). The scores for these items were averaged to create a reliable measure of *perceived personal similarity*, with higher scores indicate greater similarity (*M* = 4.02, *SD* = 1.70, α = .91).

**Table 1. Correlations between variables (N = 118; Study 1)**

|  | PATH | ATLG | TBAG&GI | PC | PIGD | PIPS |
| --- | --- | --- | --- | --- | --- | --- |
| Religiosity | -.161 | .124 | .210^*^ | .043 | -.050 | -.072 |
| Positive Attitude Towards Homosexuality (PATH) |  | -.859^**^ | -.820^**^ | -.317^**^ | -.578^**^ | .614^**^ |
| Attitude towards Lesbians and Gay men (ATLG) |  |  | .769^**^ | .308^**^ | .489^**^ | -.486^**^ |
| Traditional Beliefs about Gender and Gender Identity (TBAG&GI) |  |  |  | .287^**^ | .554^**^ | -.546^**^ |
| Perceived Control (PC) |  |  |  |  | .123 | -.267^**^ |
| Perceived Inter-Group Differences (PIGD) |  |  |  |  |  | -.715^**^ |
| Perceived Inter-Personal Similarity (PIPS) |  |  |  |  |  | - |

* *p* < .05, ** *p* < .01 (2-tailed)

**Results**

As for the main dependent variables described in the main document, these complementary measures were submitted to an ANCOVA in which we introduced participants’ religiosity (standardized scores), the experimental condition (*biological bases*: -1 = differences, +1 = similarities) and the interaction between these two factors as covariates.

*Perceived intergroup differences.* The main ANCOVA only revealed a significant interaction effect, *F*(1,114) = 4.98, *p* = .028, η_p_^2^ = .042 (Figure 2). Despite the pattern of this interaction was in the predicted direction, none of the slope analyses was significant: When religiosity was low (-1*SD*), the experimental manipulation did not influence perceived intergroup differences, *t*(114) = 1.19, *p* = .233, η^2^_p_ = .012. However, when religiosity was high (+1*SD*), perceived intergroup differences was higher in the biological differences condition than in the biological similarities condition, *t*(114) = 1.97, *p* = .050, η^2^_p_ = .033. Religiosity was not associated with perceived intergroup differences in the biological similarities condition (*B* = -.38, *SE* = .21), *t*(114) = 1.75, *p* = .082, η^2^_p_ = .026, nor in the biological differences condition (*B* = .37, *SE* = .26), *t*(114) = 1.44, *p* > .15, η^2^_p_ = .01.

*Traditional Beliefs about Gender and Gender Identity.* We initially ran a mixed ANCOVA in which the two subscales (TBG and TBGI) were introduced as a between-subjects factor. This analysis showed a main effect of the subscales, *F*(1,114) = 10.03, *p* = .002, η^2^_p_ = .081: Traditional beliefs were higher for TBGI than for TBG. However, none of the interactions including these subscales was significant (overall interaction: *F*(1,114) = 0.01, *p* = .93, η^2^_p_ < .001). Therefore, for simplicity reasons, we describe here the results of the analysis conducted on the overall scale TBG&GI (higher scores indicate more traditional beliefs about gender and gender identity).

This analysis revealed a significant main effect of religiosity (*B* = .30, *SE* =.09), *F*(1,114) = 9.27, *p* = .003, η_p_^2^ = .075. Religiosity was related to higher TBG&GI. Moreover, the interaction between religiosity and the biological bases was also significant, *F*(1,114) = 10.06, *p* = .002, η_p_^2^ = .081 (see Figure 2). When religiosity was low (-1*SD*), the experimental manipulation did not influence TBG&GI, *t*(114) = 1.70, *p* = .092, η^2^_p_ = .02. However, when religiosity was high (+1*SD*), TBG&GI were higher in the biological differences condition than in the biological similarities condition, *t*(114) = 2.68, *p* = .008, η^2^_p_ = .059. Religiosity was related to higher TBG&GI in the biological differences condition (*B* = .61, *SE* =.15), *t*(114) = 4.03, *p* < .001, η^2^_p_ = .12, but this relation was not significantly in the biological similarities condition (*B* = -.03, *SE* = .12), *t*(114) = 0.10, *p* = .920, η^2^_p_ < .001.

**Figure 2.** Predicted values for Traditional Beliefs about Gender and Gender Identity (TBG&GI), Perceived Inter-Group Differences (PIGD) and Perceived Inter-Personal Similarity (PIPS) as a function of religiosity (±1SD) and biological theory framing (Study 1).

*Perceived personal similarity.* The main analysis on perceived similarity revealed a significant main effect of biological bases, *F*(1,114) = 4.70, *p* = .032, η_p_^2^ = .04. Participants perceived gay men as more similar to themselves in the biological similarities condition (*M* = 4.31, *SD* = 1.72) than in the biological differences condition (*M* = 3.75, *SD* = 1.76). Moreover, the interaction was also significant, *F*(1,114) = 8.84, *p* = .004, η_p_^2^ = .072 (see Figure 2). When religiosity was low (-1*SD*), the experimental manipulation did not influence perceived similarity, *t*(114) = 0.61, *p* = .538, η^2^_p_ = .003. However, when religiosity was high (+1*SD*), perceived similarity was lower in the biological differences condition than in the biological similarities condition, *t*(114) = 3.58, *p* = .001, η^2^_p_ = .101. Religiosity was associated to a lower perceived similarity in the biological differences condition (*B* = -.73, *SE* = .74), *t*(114) = 3.05, *p* = .003, η^2^_p_ = .076, but this effect was not significant in the biological similarities condition (*B* = .19, *SE* = .20), *t*(114) = 0.97, *p* = .333, η^2^_p_ = .008.

In sum, the results regarding the complementary measures assessing perceived interpersonal and intergroup differences also provided evidence in support of the main hypothesis. Across three differentiation measures, participants with stronger religiosity interpreted the evidence supporting the BTSO as indicative of fundamental differences between heterosexual and gay men.

**STUDY 2**

**Dependent variables**. In addition of the materials described in the main document, Study 2 also included three additional measures for exploratory purposes: a measure of morality and two the scales included in Study 1 assessing psychological differentiation.

*Morality*. At pre-test, we also included a self-made 3-item measure assessing the *degree of morality attributed to homosexuality* (e.g., ‘Compared to my personal values, homosexuality is immoral’). This measure was included for exploratory purposes in order to examine whether religiosity was related to the morality attributed to homosexuality, and whether religiosity moderates the effect of exposure to BTSO evidence over and above participants’ perceived morality. However, it is worth noting that measures of sexual prejudice actually include items related to the perceived morality of homosexuality and sexual minorities as indicators or individuals’ attitudes towards them. Since our 3-item scale focused on homosexuality, it was not an appropriate measure to examine the specific contribution of religiosity and morality in the investigated processes, and future research should use a measure of morality that is not related to homosexuality. Therefore, for simplicity and length issues, we decided not to describe this measure in the main document.

*Perceived intergroup differences.* We used the same 3 items used in Study 1 and added two additional ones (‘Heterosexual and gay people are essentially different’ and ‘Heterosexual and gay people are psychologically different’) to obtain a 5-item scale: (*M* = 2.92, *SD* = 1.55, α = .87).

**Table 2. Correlations between variables (N= 280; Study 2).**

|  | PATH | PBA | PC | PIGD | PIPS | RDH |
| --- | --- | --- | --- | --- | --- | --- |
| Religiosity | -.296^**^ | .316^**^ | .194^**^ | .061 | -.058 | -.054 |
| Positive Attitude Towards Homosexuality (PATH) |  | -.787^**^ | -.432^**^ | -.623^**^ | .606^**^ | -.073 |
| Perceived Biological Anomaly (PBA) |  |  | .441^**^ | .688^**^ | -.559^**^ | .114 |
| Perceived Control (PC) |  |  |  | .378^**^ | -.294^**^ | .062 |
| Perceived Inter-Group Differences (PIGD) |  |  |  |  | -.590^**^ | .098 |
| Perceived Inter-Personal Similarity (PIPS) |  |  |  |  |  | -.167** |
| Relative Dehumanization (RDH) |  |  |  |  |  | - |

* *p* < .05, ** *p* < .01 (2-tailed)

*Perceived interpersonal similarity*. Participants’ perceived similarity between themselves and gay men and lesbians was assessed using a 7-item scale similar to that used in Study 1. Participants were asked to indicate how similar they thought they were to gay men or lesbians (sex-matched) in various areas, including emotional level, needs, whishes, intimate relationships, friendships, professional relationships, and in general (1 ‘*strongly different*’ to 7 ‘*strongly similar’*). Scores for these 7 items were averaged to create a reliable measure of *perceived personal similarity*, with higher scores indicating greater perceived similarity between participants and either gay men or lesbians (α = .92, *M* = 5.16, *SD* = 1.54).

*Relative Dehumanization*. At post-test, we also included a measure of intergroup dehumanization as full dependent variable. Indeed, in this study we aimed at exploring whether the investigated processes also influenced participants’ relative dehumanization of gay people. Because this measure introduced some important methodological limitations, we decided not to include it the main document. However, the results regarding this variable are described in this Supplementary Material for transparency and manuscript length concerns.

We used a measure of dehumanization that was adapted from Fasoli and colleagues (Fasoli et al., 2016). Participants received a list 10 human-related and 10 animal-related words (randomized order) and were asked to choose the 8-10 words they considered the most representative of both gay and heterosexual people (randomized order). A score of dehumanization for each group was computed by subtracting the number of human-related words from the number of animal-related words that were chosen (higher values indicate higher levels of animalistic dehumanization). Finally, we computed an overall score of relative dehumanization of gay people compared to heterosexual people (i.e., higher scores mean greater dehumanization of gay people, as compared to heterosexual people; *M* = -0.56, *SD* = 2.52).

**Results**

These complementary measures were analyzed as a function of the same analytical strategy used for the main dependent variables (see main document). A first contrast (C1) opposed the biological differences condition (coded as +2) to the other two conditions (each one coded as -1). The second residual contrast (C2) opposed the biological similarities condition (+1) to the control condition (-1), with the biological differences condition coded as 0. The ANCOVA tested the effect these two contrasts, religiosity (standardized scores), participant’s sex (-1 = women and +1 = men), and the interactions between these three factors (interactions including the two contrasts were not included). All effects were introduced as covariates.

*Perceived intergroup differences****.*** The ANCOVA revealed a significant main effect of participant’s sex, *F*(1,268) = 21.56, *p* = .001, η^2^_p_ = .074. Male participants (*M* = 3.50, *SD* = 1.65) perceived more intergroup differences than female participants (*M* = 2.66, *SD* = 1.43). The analysis also showed a significant effect of C1, *F*(1,268) = 7.59, *p* = .006, η^2^_p_ = .028. Perceived intergroup differences were higher in the biological differences condition (*M* = 3.21, *SD* = 1.69) than in the biological similarities (*M* = 2.83, *SD* = 1.45) and control (*M* = 2.72, *SD* = 1.47) conditions. These two last conditions did not differ significantly (C2), *F*(1,268) = 0.22, *p* = .63, η^2^_p_ = .001. No other effects were significant.

*Perceived personal similarity****.*** The main ANCOVA on perceived similarity only showed a significant main effect of participant’s sex, *F*(1,268) = 21.80, *p* < .001, η^2^_p_ = .075. Male participants (*M* = 4.56, *SD* = 1.66) perceived themselves as less similar to gay men than female participants did to lesbian women (*M* = 5.44, *SD* = 1.40). No other effects were significant. Thus, these results are not consistent with those observed in Study 1.

In sum, regarding these three measures of differentiation, male participants, in contrast to female participants, perceived greater intergroup differences and less personal similarity with gay individuals. Additionally, perceived intergroup differences were higher in the biological differences condition, suggesting that BTSO evidence not only impacts the naturalness dimension of essentialism, but also the discreetness or entitativity dimension (Haslam & Levy, 2006). However, this pattern was not moderated by participant’s sex or religiosity, which contrasts with the results observed in Study 1. These findings were unexpected, especially considering the effort made in Study 2 to improve the validity of these scales compared to those used in Study 1.

***Dehumanization***. Preliminary exploratory analyses identified 32 outliers on the scores of dehumanization for each group, but we decided not to remove them from the analyses given their large frequency. In addition, participants overall attributed more human-related words than animal-related words to both groups, and dehumanization was even lower for gay people (*M* = -3.51, *SD* = 2.66) than for heterosexual people (*M* = -2.94, *SD* = 2.52), *t*(279) = 3.75, *p* < .001. Furthermore, only 6.1% of participants dehumanized gay people, and only 15% of participants relatively dehumanized gay people more than heterosexual people. These results could be explained by the fact that this is measure of dehumanization is likely to be subjected to socially desirable responding effects—i.e., participants are likely to be aware of the social norms around sexual orientation being a socially protected group and thus may be unable or unwilling to report explicit dehumanization of this group.

Despite these methodological concerns, and for exploratory and transparency purposes, we conducted the main ANCOVA used in Study 2 (main document) on the relative dehumanization of gay people compared to heterosexual people. The interaction between C1 and religious beliefs felt short of significance, *F*(1,268) = 3.79, *p* = .053, η^2^_p_ = .014, but that the overall interaction between C1, religious beliefs and participant gender was significant, *F*(1,268) = 3.92, *p* = .048, η^2^_p_ = .014 (Figure 3).

**Figure 3.** Predicted values for the relative dehumanization of gay people (as compared to heterosexual people) as a function of religious beliefs (±1SD), biological theory framing and participant gender (Study 2).

No significant effects were observed among female participants, *F*s < 1.75, *p*s > .18. However, among male participants, the interaction between C1 and religious beliefs was significant, *F*(1,268) = 4.85, *p* = .028, η^2^_p_ = .018. Religious beliefs tended to be associated with greater relative dehumanization of gay people in the biological differences condition (*B* = 1.23), *t*(268) = 1.94, *p* = .053, η^2^_p_ = .014, and to a lower relative dehumanization in the control condition (*B* = -.77), *t*(268) = 1.72, *p* = .086, η^2^_p_ = .011, but this relationship was far from significance in the biological similarities condition (*B* = .06), *t*(268) = 0.12, *p* = .89, η^2^_p_ < .001. We also decomposed the interaction the other way around. The effect of C1 was significant when religious beliefs were relatively weak (-1*SD*; *B* = .57), *t*(268) = 1.97, *p* = .05, η^2^_p_ = .014: male participants with low religious beliefs dehumanized gay people (as compared to heterosexual people) to a lesser extent in the biological differences condition, as compared to the biological similarities condition. However, when religious beliefs were strong (+1*SD*) the effect of C1 was not significant (*B* = .49), *t*(268) = 1.47, *p* = .14, η^2^_p_ = .008.

Despite these findings are consistent with our predictions, and with the results observed as a function of participant’s gender, the observed effects were relatively weak and the measure of explicit dehumanization of gay people had important methodological limitations. Therefore, further research is needed to examine more appropriately whether our main hypothesis also generalizes to explicit and implicit measures of dehumanization.

**References**

Dasgupta, N., & Rivera, L. M. (2006). From automatic antigay prejudice to behavior: The moderating role of conscious beliefs about gender and behavioral control. *Journal of Personality and Social Psychology*, *91*(2), 268–280. https://doi.org/10.1037/0022-3514.91.2.268

Falomir‐Pichastor, J. M., & Hegarty, P. (2014). Maintaining distinctions under threat: Heterosexual men endorse the biological theory of sexuality when equality is the norm. *British Journal of Social Psychology*, *53*(4), 731–751. https://doi.org/10.1111/bjso.12051

Falomir-Pichastor, J. M., & Mugny, G. (2009). “I’m not gay. . . . I’m a real man!”: Heterosexual Men’s Gender Self-Esteem and Sexual Prejudice. *Personality and Social Psychology Bulletin*, *35*(9), 1233–1243. https://doi.org/10.1177/0146167209338072

Fasoli, F., Paladino, M. P., Carnaghi, A., Jetten, J., Bastian, B., & Bain, P. G. (2016). Not “just words”: Exposure to homophobic epithets leads to dehumanizing and physical distancing from gay men. *European Journal of Social Psychology*, *46*(2), 237–248. https://doi.org/10.1002/ejsp.2148

Haslam, N., & Levy, S. R. (2006). Essentialist Beliefs About Homosexuality: Structure and Implications for Prejudice. *Personality and Social Psychology Bulletin*, *32*(4), 471–485. https://doi.org/10.1177/0146167205276516

Thompson, E. H., & Pleck, J. H. (1986). The Structure of Male Role Norms. *American Behavioral Scientist*, *29*(5), 531–543. https://doi.org/10.1177/000276486029005003

1. These two master dissertations were conducted independently, had different goals and materials that are beyond the scope of this pilot study, and are not described here. Therefore, we only describe here the parts of the material that fit the scope of the present research. More information about these specific goals and materials can be obtained from the first author. [↑](#footnote-ref-1)
2. We are grateful to Amandine Friedman and Annie Chemla, who recruited participants in the realm of their master dissertation. The two studies were conducted separately and independently, and therefore they included different goals and additional materials that are not described here. [↑](#footnote-ref-2)
3. The same analysis was also conducted whilst additionally including participant’s gender. Gender main effect was significant, *F*(1,176) = 12.39, *p* = .001, η_p_^2^ = .067, indicating that female participants (*M* = 5.55, *SD* = 1.09) showed more positive attitudes than male participants (*M* = 4.83, *SD* = 1.08). However, none of the interaction effects including participant’s gender were significant, *F*s < 1.37, *p*s > .24. Given that female participants (*n* = 47) were underrepresented in the present sample, and that analyses including gender as an additional predictor were underpowered, we decided not to take participants gender into consideration in these analyses. [↑](#footnote-ref-3)
